# NDK Interacts with FtsZ and Converts GDP to GTP to Trigger FtsZ Polymerisation - A Novel Role for NDK

**DOI:** 10.1371/journal.pone.0143677

**Published:** 2015-12-02

**Authors:** Saurabh Mishra, Kishor Jakkala, Ramanujam Srinivasan, Muthu Arumugam, Raghavendra Ranjeri, Prabuddha Gupta, Haryadi Rajeswari, Parthasarathi Ajitkumar

**Affiliations:** Department of Microbiology and Cell Biology, Indian Institute of Science, Bangalore, India; University of Groningen, Groningen Institute for Biomolecular Sciences and Biotechnology, NETHERLANDS

## Abstract

**Introduction:**

Nucleoside diphosphate kinase (NDK), conserved across bacteria to humans, synthesises NTP from NDP and ATP. The eukaryotic homologue, the NDPK, uses ATP to phosphorylate the tubulin-bound GDP to GTP for tubulin polymerisation. The bacterial cytokinetic protein FtsZ, which is the tubulin homologue, also uses GTP for polymerisation. Therefore, we examined whether NDK can interact with FtsZ to convert FtsZ-bound GDP and/or free GDP to GTP to trigger FtsZ polymerisation.

**Methods:**

Recombinant and native NDK and FtsZ proteins of *Mycobacterium smegmatis* and *Mycobacterium tuberculosis* were used as the experimental samples. FtsZ polymersation was monitored using 90° light scattering and FtsZ polymer pelleting assays. The γ^32^P-GTP synthesised by NDK from GDP and γ^32^P-ATP was detected using thin layer chromatography and quantitated using phosphorimager. The FtsZ bound ^32^P-GTP was quantitated using phosphorimager, after UV-crosslinking, followed by SDS-PAGE. The NDK-FtsZ interaction was determined using Ni^2+^-NTA-pulldown assay and co-immunoprecipitation of the recombinant and native proteins *in vitro* and *ex vivo*, respectively.

**Results:**

NDK triggered instantaneous polymerisation of GDP-precharged recombinant FtsZ in the presence of ATP, similar to the polymerisation of recombinant FtsZ (not GDP-precharged) upon the direct addition of GTP. Similarly, NDK triggered polymerisation of recombinant FtsZ (not GDP-precharged) in the presence of free GDP and ATP as well. Mutant NDK, partially deficient in GTP synthesis from ATP and GDP, triggered low level of polymerisation of MsFtsZ, but not of MtFtsZ. As characteristic of NDK’s NTP substrate non-specificity, it used CTP, TTP, and UTP also to convert GDP to GTP, to trigger FtsZ polymerisation. The NDK of one mycobacterial species could trigger the polymerisation of the FtsZ of another mycobacterial species. Both the recombinant and the native NDK and FtsZ showed interaction with each other *in vitro* and *ex vivo*, alluding to the possibility of direct phosphorylation of FtsZ-bound GDP by NDK.

**Conclusion:**

Irrespective of the bacterial species, NDK interacts with FtsZ *in vitro* and *ex vivo* and, through the synthesis of GTP from FtsZ-bound GDP and/or free GDP, and ATP (CTP/TTP/UTP), triggers FtsZ polymerisation. The possible biological context of this novel activity of NDK is presented.

## Introduction

Nucleoside diphosphate kinase (NDK) (EC 2.7.4.6), called NDPK in eukaryotes, was discovered simultaneously but independently by Sir Hans Krebs [1] and Paul Berg [2]. It synthesises nucleoside triphosphates (NTPs) by transferring the 5’ terminal phosphate from ATP or GTP to nucleoside diphosphates (NDPs) [3–8]. During the process of the transfer, the NDKs form a high energy phosphate intermediate on the histidine residue at the active site of the enzyme [3–8]. NDK/NDPK is widely conserved across all the three domains of life, namely eubacteria, archaea, and eukarya (reviewed in [9–11]). The NDK of *Mycobacterium tuberculosis* (MtNDK) and of *Mycobacterium smegmatis* (MsNDK) have been biochemically characterised [12–14]. The three-dimensional hexameric structure of MtNDK has been solved [15] and the intersubunit interactions among its six subunits has been elucidated [14]. While the active form of both MtNDK and MsNDK are hexamers, they also exist as dimers and tetramers [12–15]. They have comparable biochemical characteristics, having His-117 at the active site [12–15]. The H117Q mutation almost abolishes the phosphotransfer activity, leaving residual activity [12,13].

NDKs are substrate non-specific enzymes, as they can utilise different NTPs as their source of phosphate for their phosphate transfer activity [5]. The activity of NDKs can also be directed towards the synthesis of any specific NTP by specific proteins that need the particular NTP for their function. For example, in *M*. *smegmatis*, P_70_ (pyruvate kinase) and P_50_ (homologous to elongation factor EF-Tu) can alter the specificity of MsNDK towards GTP formation, as these proteins utilise GTP [16]. Further, while the CTP-requiring P_60_ (cell wall antigen) can alter its specificity towards CTP synthesis, the UTP-requiring P_65_ modulates its specificity towards UTP synthesis [16]. *Pseudomonas aeruginosa* NDK exists as a 16 kDa cytoplasmic form and as a 12 kDa truncated membrane-associated form, both of which form a complex with succinyl coenzyme A synthetase (SuCoAs) [17]. The 16 kDa NDK-SuCoAs complex, which is present in the nonmucoid cells and predominant in the log phase, synthesises GTP, UTP, and CTP. On the contrary, the 12 kDa NDK-SuCoAs complex present in the mucoid cells and predominant in the stationary phase synthesises only GTP and UTP, but not CTP [17]. These observations show that specific interacting proteins can direct NDK to synthesise specific NTPs that are required for the function of those interacting proteins, as demanded by the cellular needs.

NDK/NDPK has been found to phosphorylate GDP, which is bound to G proteins, to GTP and thereby activate the G proteins [18–25]. NDPK binds succinate thiokinase, and channels high energy phosphate from GTP produced in the Krebs cycle to the ATP pool, which in turn accounts for the ATP-supported succinyl-CoA synthetase activity [26]. NDPK can act as a source of GTP for the elongation factor Tu (EF-Tu) during protein biosynthesis [27] and for Dynamin-dependent synaptic vesicle recycling [28]. Recently, human NDPKs have been found to interact with dynamins and provide GTP for membrane remodeling [29]. Mammalian NDPKs interact with diverse cytoskeletal components and regulators, such as actin-binding proteins, intermediate filaments, and cytoskeletal attachment structures (adherens junctions, desmosomes, and focal adhesions) (reviewed in [30]).

NDPK was found associated with the tubulin purified from the brain cell lysates of guinea pig [31–33], chicken [34], and bovine [35]. Further, NDPK assists tubulin polymerisation by converting tubulin-bound GDP to GTP in the presence of nucleotides *in vitro* [31–33]. The bacterial homologue of tubulin, bacterial cytokinetic and cytoskeletal protein, FtsZ [36,37], binds GTP [38–40] and undergoes GTP-dependent polymerisation [41,42], like tubulin [36,37]. Since there is striking conservation of NDKs/NDPKs across the living kingdom [9–11] and strong structural and functional homology between FtsZ and tubulin [36,37], the present study was designed to find out whether bacterial NDK can specifically synthesise GTP, from ATP and FtsZ-bound GDP or free GDP, to trigger FtsZ polymerisation. If it can, whether NDK would physically interact with FtsZ during the process.

The recombinant *M*. *smegmatis* FtsZ (MsFtsZ) [43] and *M*. *tuberculosis* FtsZ (MtFtsZ) [44,45] and the respective MsNDK [13] and MtNDK [12] were used as the experimental system for the following reasons. We wanted to investigate the possible role of NDK in FtsZ function, as part of the ongoing studies on the functional modulation of mycobacterial FtsZ [43–45] in cell division. Further, while FtsZ is a potential antibacterial drug target [46], NDK is the hub of a metabolic network in mycobacteria [47]. Therefore, the possibility of an interaction between FtsZ and NDK, to control the cytokinetic and cytoskeletal functions of the essential protein FtsZ, would offer scope to target the interaction for chemotherapeutic intervention against Tuberculosis.

The ability of NDK to trigger FtsZ polymerisation was determined by monitoring the FtsZ polymer formation, using 90° light scattering assay, and FtsZ polymer pelleting assay. The production of GTP by NDK, from GDP and γ^32^P-ATP to γ^32^P-GTP and ADP for the polymerisation of FtsZ, was monitored using PEI-cellulose thin layer chromatography. The morphology of the FtsZ polymers, formed from the NDK-triggered FtsZ polymerisation was compared with the FtsZ polymers formed from the direct addition of GTP, using transmission electron microscopy. The physical interaction between recombinant and native FtsZ and NDK was determined *in vitro* and *ex vivo* using pulldown assay and co-immunoprecipitation assay. The present study reveals physical interaction of NDK with FtsZ, conserved across bacterial genera, to trigger FtsZ polymerisation by phosphorylating FtsZ-bound GDP or free GDP, using ATP or CTP/TTP/UTP as the phosphate donor.

## Materials and Methods

### Bacterial Strains

The bacterial strains used in the study are listed in S1 Table. *Escherichia coli* strains were grown in Luria Broth (Difco) at 30°C or at 37°C. *M*. *tuberculosis* H_37_R_a_ (kind gift from JALMA Institute of Leprosy and Other Mycobacterial Diseases, Agra) and *M*. *smegmatis* mc^2^155 [48] cells were cultured in Middlebrook 7H9 (Difco) liquid medium supplemented with enrichment or in Middlebrook 7H10 agar (Difco) medium supplemented with enrichment.

### Construction of Plasmid Vectors and Overexpression and Purification of Proteins

The primers used for the cloning of the genes are listed in S2 Table and the plasmid vectors (maintained in *E*. *coli* JM109 [49]) used/or generated in this study are listed in S3 Table. The construction of the plasmid vectors used in this study and overexpression, purification of MsFtsZ and MtFtsZ proteins and MsNDK, MtNDK and their H117Q mutants, and production of polyclonal antibodies and their immunoaffinity purification are given under S1 Text.

### Preparation of GDP-Precharged FtsZ and GDP-Depleted FtsZ

About 2000 μg of FtsZ protein was incubated with 1 mM of GDP (final concentration) in 200 μl volume for 3 hrs at 4°C. Then this mix was loaded on G-25 column (pre-equilibrated with 50 mM HEPES-NaOH buffer, pH 7.2 containing 1mM DTT and 10% glycerol. Column was centrifuged repeatedly at 1000 g for 1 min at 4°C to collect eluted fractions to separate protein from free GDP. Most of the protein was found to be in 1st fraction. GDP-free FtsZ was prepared, as described [50], with the following modifications. About 2000 μg of FtsZ protein was incubated with 2.5 M guanidinium hydrochloride (GdmHCl) for 30 min at room temperature, followed by gel filtration in Sephadex G-25 column (pre-equilibrated with 50 mM HEPES-NaOH buffer, pH 7.2 containing 1 mM DTT, 10% glycerol and 2.5 M GdmHCl) to separate the protein from the released nucleotide. Eluted protein was step-dialysed to remove GdmHCl.

### 90° Light Scattering (LS) Assay for FtsZ Polymerisation

FtsZ polymerisation was monitored using 90° light scattering assay [43–45,51,52] using FluoroMax-4 spectrofluorimeter. In the 90° light scattering assay, if l\λ > 3.5, where l = length of the protein polymer, λ = wavelength of incident light, then 90° scattered light by the polymer is proportional to polymer weight and independent of length distribution [53]. Protein samples were taken in 2-(N-morpholino)ethanesulfonic acid-NaOH (MES-NaOH) (pH 6.5) buffer containing 50 mM KCl and 5 mM MgCl_2_ in a reaction volume of 150 μl. The polymerisation was carried out at 2.5 mM Mg^2+^ concentration also. The concentration of FtsZ and NDK were 8.6 μM and 0.1 μM, respectively. The protein samples were incubated, in the absence of NTP, with or without 1 mM GDP, initially for 200 sec, at 30°C, to get a baseline. Polymerisation was triggered with the addition of 1 mM of NTPs (GTP, ATP, CTP, TTP, or UTP). Reactions were monitored for further 400 or 600 sec. The data acquisition interval was set as 40 sec, which was the minimum time required for the addition and mixing of the NTP with the polymerisation reaction mixture.

### FtsZ Polymer Pelleting Assay

In the FtsZ polymer pelleting assay [45,54], polymerisation was performed with GTP, or GDP and ATP (or other NTPs). The polymerised protein was estimated using ultrasedimentation, as described [54], with minor modifications [45]. The polymerisation conditions were identical to that used for the 90° light scattering assay, except that the reaction volume was 200 μl. Reactions were initiated with the addition of 5 mM GTP or 5 mM of other NTPs (ATP, CTP, TTP, or UTP), with or without GDP, and incubated at 30°C for 10 min. Immediately after the completion of the polymerisation reaction, the samples were centrifuged at 80000 rpm (247000 x g) at 4°C in Beckman TLA100 rotor (180 μl in 200 μl ultracentrifuge tube; with maximum force of 436000 x g and minimum force of 336000 x g for 100000 rpm), in OptimaTM TLX ultracentrifuge, for 10 min. After centrifugation, the supernatant was carefully transferred to fresh microcentrifuge tube and the pellet was resuspended in 100 μl of 1x SDS-PAGE loading buffer. One-tenth the quantity of the supernatant and the pellet fractions was loaded onto SDS-PAGE.

### Transmission Electron Microscopy (TEM)

TEM was performed, as described [44,45]. The polymerisation conditions were identical to that used for the 90° light scattering assay, except that the Mg^2+^ concentration used was always 5 mM. The polymerised samples were visualised under JEOL JEM 100 CX II or FEI Tecnai BioTWIN transmission electron microscope. All the TEM pictures were taken at different magnifications, namely 14000 or 20000 (with/without further digital magnification in JEOL), or at 105000 in FEI Tecnai BioTWIN.

### γ^32^P-GTP Formation Assay

The proteins samples (FtsZ or NDK alone or FtsZ and NDK together) were taken in MES-NaOH (pH 6.5) buffer containing 50 mM KCl and 5 mM MgCl_2_ in a reaction volume of 20 μl. The concentration of FtsZ was maintained at 8.6 μM [44,45] and of NDK at 0.1 μM [13], along with 1 mM GDP. The reactions were initiated with the addition of γ^32^P-ATP with or without cold nucleotides, incubated at 30°C for 10 min, and terminated with the addition of 25 mM final concentration of EDTA followed by incubation at 75°C for 5 min. After giving a brief spin, 1 μl of supernatant was loaded on PEI-cellulose TLC sheets and the nucleotides were resolved in 0.75 M KH_2_PO_4_ [55]. The radiolabelled nucleotide spots were visualised using phosphorimager and quantitated densitometrically using Multi-Gauge software.

### Assay for γ^32^P-GTP Formation And Its Binding to FtsZ

The assay for γ^32^P-GTP binding to FtsZ was performed as described [31,38,39], with the following modifications. The reactions were set up exactly as described under GTP formation assay, but were incubated initially for 3 min at 30°C, followed by the addition of radio nucleotides (γ^32^P-ATP or γ^32^P-GTP) with or without cold nucleotides. The reactions were performed for 30 sec, stopped with the addition of 25 mM EDTA, and immediately divided into two parts. One part was used for the assay of γ^32^P-GTP formation and the other part was used for the assay of γ^32^P-GTP binding to FtsZ. For the γ^32^P-GTP formation assay, an equal volume of 95% ethanol was added to the reaction mixture, chilled in liquid N_2_, centrifuged at 12000 g at 4°C, one μl of the supernatant was spotted on PEI cellulose sheets, and resolved using 0.75 M KH_2_PO_4_ [55]. The radiolabelled nucleotide spots were visualised using phosphorimager and quantitated densitometrically using Multi-Gauge software.

The second part of the reaction mentioned above was kept on ice and exposed to UV in Hoefer UVC 500 ultraviolet crosslinker at 200000 μJ/cm2 for 5 min. Immediately after the UV cross-linking, the protein samples were resolved on 12% SDS polyacrylamide gel. The bands corresponding to the cross-linked γ^32^P-GTP-FtsZ protein were visualised using phosphorimager and quantitated using densitometric principles with Multi-Gauge. The values were normalised with respect to the ethanol-extracted total γ^32^P-GTP in the reaction. Direct binding of α^32^P-GTP to FtsZ was used as the positive control.

### Ni^2+^-NTA-Agarose Pulldown Assay

The pull-down assays using purified 6xHis-tagged or GST-tagged recombinant proteins were carried out in the presence of the cross-linking agent, 3,3’-dithio-bis (N-hydroxysuccinimidyl propionate) [DTSP], essentially as described [56]. Five μM concentration of purified proteins were mixed with 5 μM BSA in Hyb buffer [25 mM HEPES-KOH (pH 7.7), 50 mM KCl, 0.1 mM EDTA, 2.5 mM MgCl_2_, 1 mM DTT, 1% nonfat dried milk powder and 0.05% NP-40], 250 μM of DTSP was added, and incubated at 30°C for 30 min. 1 M Tris-HCl (pH 7.4) buffer was then added to a final concentration of 20 mM to quench the reaction. The reaction mixture was further incubated for 15 min at 30°C. Fifty μl of Ni^2+^-NTA-agarose slurry, equilibrated with Hyb buffer, was mixed with the above solution and incubated at 4°C overnight with continuous mixing. The beads were then washed 5 times with 1 ml PBS containing 0.05% NP-40 and 20 mM imidazole. The protein was then eluted from the beads by incubating with 50 mM Tris-HCl (pH 8.0) containing 250 mM NaCl and 250 mM imidazole. The eluate was resolved on 12% SDS-PAGE, and immunoblotted (see S1 Text).

### Co-Immunoprecipitation of MsNDK and MsFtsZ

Coimmunoprecipitation was performed using purified recombinant MsNDK and MsFtsZ purified proteins (10 μg each) or *M*. *smegmatis* mc^2^155 whole cell lysate (300 μg). The samples were incubated for 3 hrs at 4°C in TK buffer (100 mM Tris-HCl, pH 8.0, and 50 mM KCl) with affinity-purified anti-MsNDK or anti-MsFtsZ polyclonal antibody. About 25 μl of protein A sepharose beads were added to the co-immunoprecipitate, incubated for 3 hrs at 4°C, washed with TK buffer to remove unbound proteins, and collected by brief centrifugation. The beads containing protein complex were incubated in 1x SDS loading buffer for 10 min at 95°C. The eluted proteins were resolved on 12% SDS-PAGE, electro-transferred to PVDF membrane, and probed with anti-MsNDK or anti-MsFtsZ antibody for 3 hrs at 4°C. After three washes with wash buffer (1xPBS containing 1% fat-free dried milk powder and 0.05% NP40), incubated with anti-rabbit HRP secondary antibody (Sigma) for 2 hrs at 4°C, washed, and developed using ECL reagent (Sigma).

## Results

### NDK Uses GDP and ATP to Trigger FtsZ Polymerisation

**Fig 1:**
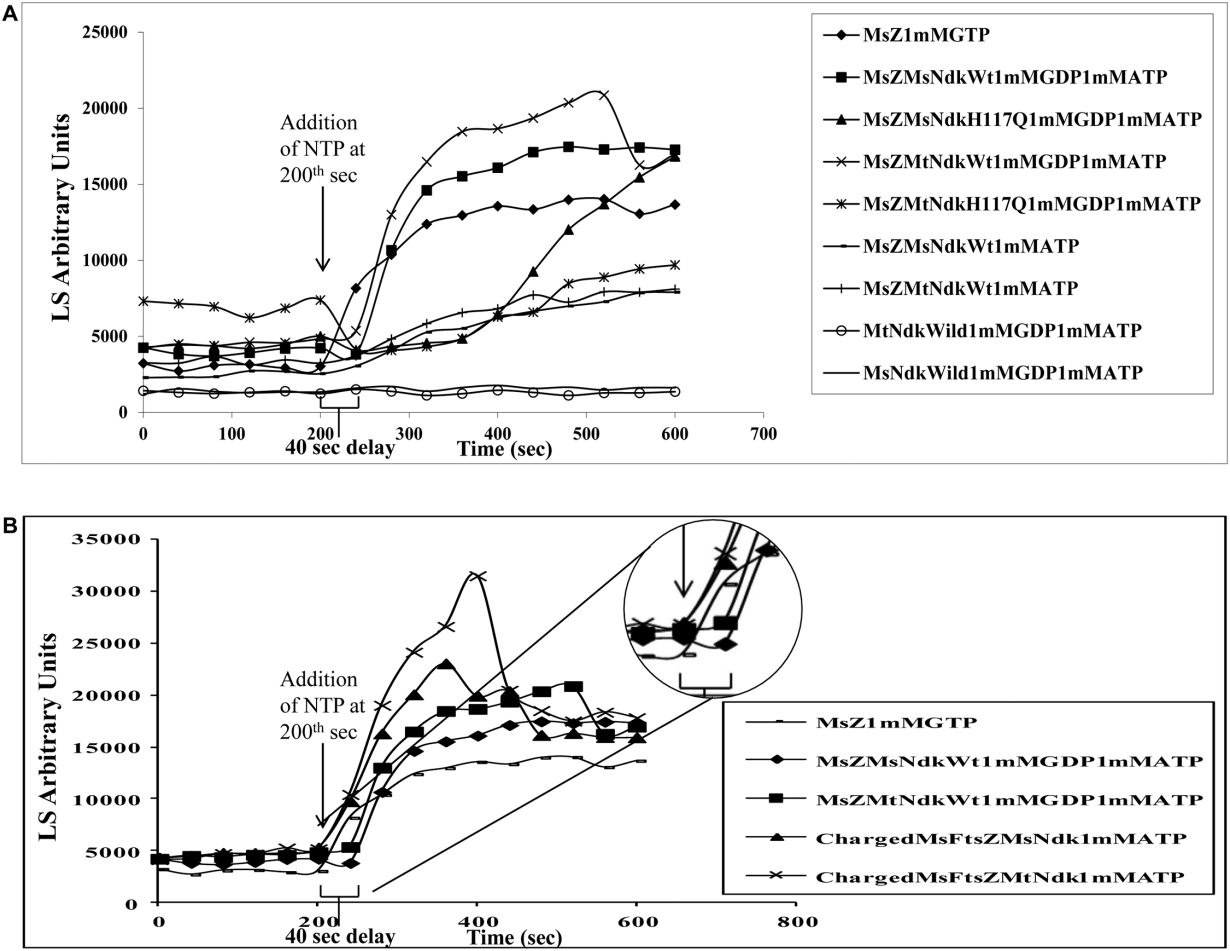
90° Light Scattering (LS) assay profiles of MsFtsZ polymerisation. Polymerisation in the presence of exogenous GTP or in the presence of exogenous GDP, ATP, and MsNDK or MtNDK (wild type or mutant). (**A**) Polymerisation of recombinant purified MsFtsZ. (**B**) Polymerisation of recombinant purified GDP-precharged MsFtsZ. The notations for the different samples, their positive and negative control samples are given in the inset text. GTP/ATP was added at the 200^th^ sec, after developing a base line, to trigger polymerisation. The 40 sec delay in the polymerisation is indicated using a square bracket placed close to the X-axis and also depicted in the inset of the enlarged portion. Note the absence of the 40 sec delay in the case of the direct addition of GTP. The MsZ1mMGTP, MsZMsNdkWt1mMGDP1mMATP, and MsZMtNdkWt1mMGDP1mMATP reactions are common to A & B.

Addition of 1 mM GTP at the 200^th^ sec (after generating a baseline) caused instant polymerisation of 8.6 μM purified MsFtsZ, without any delay in the LS assay (Fig 1A, ♦ symbol), as expected of purified mycobacterial FtsZ protein [44,45,52]. Polymerisation of MsFtsZ could also be triggered by 0.1 μM MsNDK, without the direct addition of GTP, but in the presence of 1 mM GDP and 1 mM ATP (Fig 1A, ■ symbol). However, unlike the instant polymerisation upon the direct addition of GTP, a delay of about 40 sec post-addition of ATP (ATP was added at the 200^th^ sec), could be observed (Fig 1A, ■ symbol; see the delay shown by the demarcation on the X-axis on the graph). Forty second was the minimum time required to add GTP/ATP to the reaction mixture and hence 40 sec was kept as the data acquisition interval for polymerisation. The cross-species NDK, MtNDK, also triggered polymerisation of MsFtsZ in the presence of 1 mM GDP and 1 mM ATP, again with a delay of about 40 sec post ATP addition (ATP was added at the 200^th^ sec), as revealed by the LS assay (Fig 1A, × symbol). Comparable extent of light scattering amongst the three reactions (Fig 1A, ♦, ■, and × symbols) indicated that the mass of the FtsZ polymers formed was comparable, as per the principle of 90° light scattering [53]. The polymerisation of MsFtsZ in the presence of 2.5 mM Mg^2+^, was comparable to the polymerisation in the presence of 5 mM Mg^2+^, which was used in all the experiments, as triggered by GTP alone or by NDK in the presence of GDP and ATP (S1 Fig).

The MsNDK-H117Q and the MtNDK-H117Q mutants (negative control samples), did not trigger MsFtsZ polymerisation (Fig 1A, ▲ and җ symbols, respectively), unlike the wild type NDK protein. The delayed shallow rise of the light scattering activity may be due to the aggregation of the MsFtsZ protein over a period or due to the residual kinase activity of the mutant NDKs to convert GDP to GTP. In the absence of exogenously added GDP, but in the presence of 1 mM ATP alone, MsNDK or MtNDK could trigger only slow and shallow polymerisation of MsFtsZ, as indicated by the slow and shallow rise in the LS assay (Fig 1A, ־ and + symbols, respectively). This might be due to the NDK-mediated phosphorylation of the residual GDP remaining bound to FtsZ, as 10–40% of the purified recombinant FtsZ preparations are known to contain bound GDP [50,52,57–59]. Polymerisation did not occur without MsFtsZ, in spite of the presence of MsNDK or MtNDK, and GDP and ATP (Fig 1A,—and ○ symbols, respectively), showing that the polymerisation was FtsZ-specific.

Interestingly, MsNDK and MtNDK triggered instant polymerisation of GDP-precharged MsFtsZ, without the 40 sec delay, in the presence of ATP (Fig 1B, ▲ and × symbols, respectively), like in the case of the direct addition of GTP (Fig 1B, ־ symbol). In fact, polymerisation of GDP-precharged MsFtsZ showed a much higher level of light scattering (3–4 fold above the base line) indicative of higher polymer mass, as compared to the polymerisation caused by the direct addition of GTP (Fig 1B, compare ▲ and × symbols, with the ־ symbol). It was also a much higher extent of polymerisation than that was triggered by both the NDKs in the presence of exogenous GDP and ATP, with a delay of 40 sec in each case (Fig 1B, compare ▲ and × symbols, with the ♦ and ■ symbols). Transmission electron micrographs (TEM) showed that the morphology of the MsFtsZ polymers formed through MsNDK-triggered polymerisation (using ATP and GDP) was comparable to that of the polymers formed by the direct addition of GTP to MsFtsZ (in the absence of MsNDK) (S2 Fig). Identical observations were made in the MtFtsZ polymerisation triggered by MsNDK and MtNDK (S3 and S4 Figs; S1 Data). Thus, in the presence of ATP, MsNDK and MtNDK trigger polymerisation of GDP-precharged MsFtsZ and MtFtsZ instantaneously than it does in the presence of exogenous GDP.

### FtsZ Polymer Pelleting Assay Confirms NDK-Triggered FtsZ Polymerisation

**Fig 2:**
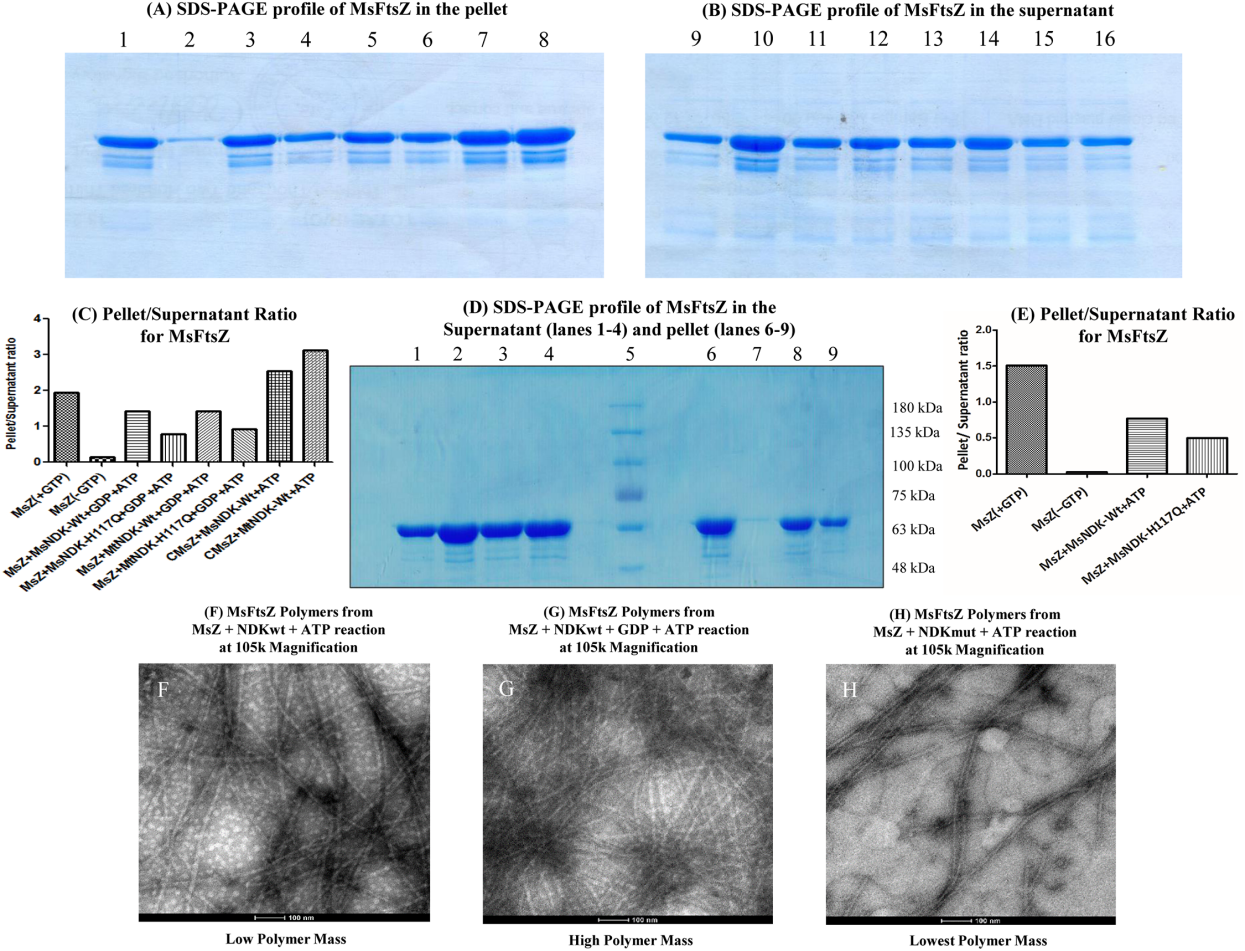
SDS-PAGE profile of the FtsZ polymer pelleting assay for MsFtsZ polymerisation and its quantitation. The SDS-PAGE profile of the sample in the pellet (**A**) and in the supernatant (**B**). Its quantitation is given as the pellet/supernatant ratio in (**C**). (**A & B**) Lanes: 1 & 9, MsFtsZ + GTP; 2 & 10, MsFtsZ without GTP; 3 & 11, MsFtsZ + MsNDK-Wt + GDP + ATP; 4 & 12, MsFtsZ + MsNDK-H117Q + GDP + ATP; 5 & 13, MsFtsZ + MtNDK-Wt + GDP + ATP; 6 & 14, MsFtsZ + MtNDK-H117Q + GDP + ATP; 7 & 15, GDP-precharged MsFtsZ + MsNDK-Wt + ATP; 8 & 16, GDP-precharged MsFtsZ + MtNDK-Wt + ATP. (**C**) Quantitations of the protein as the pellet/supernatant ratio in the reactions are represented by the bars, which are described in the figure itself. (**D**) The SDS-PAGE profile of the samples in the supernatant and pellet from MsFtsZ polymerisation in the absence of exogenous GDP. (**E**) Its quantitation given as the pellet/supernatant ratio. Lanes in (**D**): 1, MsFtsZ + GTP (Sup); 2, MsFtsZ—GTP (Sup); 3, MsFtsZ + MsNDK-Wt + ATP (Sup); 4, MsFtsZ + MsNDK-H117Q + ATP (Sup); 5, Marker; 6, MsFtsZ + GTP (Pellet); 7, MsFtsZ—GTP (Pellet); 8, MsFtsZ + MsNDK-Wt + ATP (Pellet); 9, MsFtsZ + MsNDK-H117Q + ATP (Pellet). (**E**) Quantitations of the protein as the pellet/supernatant ratio in the reactions are represented by the bars, which are described in the figure itself. (**F-H**) Comparison of TEM images (105k magnification) of FtsZ polymers formed from polymerisation of MsFtsZ triggered by MsNDKwt and MsNDKmut in the absence and presence of exogenous GDP. (**F**) MsFtsZ polymers from MsZ + NDKwt + ATP reaction; (**G**) MsFtsZ polymers from MsZ + NDKwt + GDP + ATP reaction; (**H**) MsFtsZ polymers from MsZ + NDKmut + ATP reaction. Images were captured in FEI Tecnai BioTWIN EM at 105k magnification.

The presence of higher amount of MsFtsZ in the pellet, but not in the supernatant, confirmed MsFtsZ polymerisation triggered by MsNDK or MtNDK in the presence of GDP and ATP (Fig 2A and 2B, lanes 3 & 5 and 11 & 13, respectively). The pellet to supernatant ratio of MsFtsZ reflected the presence of higher levels of polymerised MsFtsZ (Fig 2C, bars 3 & 5, respectively). In the positive control, where GTP was directly added to MsFtsZ, high levels of MsFtsZ could be observed in the pellet, but not in the supernatant (Fig 2A and 2B, lanes 1 & 9, respectively; Fig 2C, bar 1). In the absence of GTP, the entire quantity of MsFtsZ could be detected in the supernatant (negative control) (Fig 2A and 2B, compare lane 10 with lane 2; Fig 2C, bar 2). The GDP-precharged MsFtsZ showed a much higher level of polymerisation in the presence of both MsNDK and MtNDK, and 1 mM ATP, as revealed by the excess of MsFtsZ protein in the pellet (Fig 2A, lanes 7 & 8, respectively; Fig 2C, bars 7 & 8, respectively) than in the supernatant (Fig 2B, lanes 15 & 16, respectively). The MsNDK-H117Q and MtNDK-H117Q triggered only low extent of MsFtsZ polymerisation, in the presence of GDP and ATP, as revealed by the relatively low levels of MsFtsZ protein in the pellet (Fig 2A, lanes 4 & 6, respectively; Fig 2C, bars 4 & 6, respectively) and higher levels in the supernatant (Fig 2B, lanes 12 & 14, respectively). Identical observations were made in the MtFtsZ polymerisation triggered by MsNDK and MtNDK (S5 Fig; S1 Data). Thus, the FtsZ polymer pelleting assay confirmed the FtsZ polymerisation triggering activity of NDK.

The slow and shallow rise in the LS assay of the polymerisation of MsFtsZ, triggered by MsNDK and ATP, in the absence of exogenous GDP (see Fig 1A, ־ symbol) indicated formation of polymers of lower mass. Therefore, we determined the quantity of the polymer mass formed using FtsZ pelleting assay and also examined the polymers using TEM. In agreement with the slow rise in the LS assay, the pelleting assay showed that the supernatant contained larger quantity of the protein, as compared to that in the pellet (Fig 2D and 2E). It indicated that the extent of polymerisation was less in the absence of exogenous GDP. In agreement with the MsFtsZ polymer pelleting assay, transmission electron micrographs of the polymers showed lower density of the polymers (Fig 2F), as compared to the high density of MsFtsZ polymers formed in the presence of exogenous GDP (Fig 2G). The density of the polymers was very low and the polymer length was discontinuous when polymer formation was triggered by mutant NDK (Fig 2H and S2D Fig).

### NDK Uses Other NTPs In Lieu of ATP to Trigger FtsZ Polymerisation

As characteristic of the substrate non-specificity of NDKs [5], MsNDK and MtNDK could utilise other NTPs also (CTP, TTP, or UTP), in lieu of ATP, to trigger significant extent of MsFtsZ polymerisation, with the delay of about 40 sec post-addition of the NTP (S6–S8 Figs, ■ & × symbols, respectively). Both MsNDK-H117Q and MtNDK-H117Q did not support MsFtsZ polymerisation (S6–S8 Figs, ▲ and ж symbols, respectively). The positive control, MsFtsZ with 1 mM GTP, showed instant polymerisation at the addition of GTP, without any delay (S6–S8 Figs, ♦ symbol). MsNDK and MtNDK supported only low levels of polymerisation, in the absence of GDP (S6–S8 Figs, ־ and + symbols, respectively), and no polymerisation in the absence of MsFtsZ (S6–S8 Figs, ○ and—symbols, respectively). As in the case with ATP, using these NTPs (CTP, TTP, and UTP), MsNDK and MtNDK triggered instant polymerisation (without the 40 sec delay) of the GDP-precharged MsFtsZ (S9–S11 Figs, ▲ and × symbols, respectively; compare with Fig 1B, ▲ and × symbols, respectively). Again, the control samples, MsFtsZ polymerisation triggered by MsNDK or MtNDK, in the presence of exogenous GDP and other NTPs, showed the characteristic 40 sec delay (S9–S11 Figs, ♦ and ■ symbols, respectively; compare with Fig 1B, ♦ and ■ symbols, respectively). Identical observations were made in the MtFtsZ polymerisation triggered by MsNDK and MtNDK, with some deviations (S12–S14 Figs; S1 Data). Thus, true to the substrate non-specificity of NDKs, MsNDK and MtNDK could use NTPs other than ATP also for triggering FtsZ polymerisation.

### NDK Converts GDP to GTP to Trigger FtsZ Polymerisation

Triggering of instantaneous polymerisation of GDP-precharged FtsZ by NDK, without the 40 sec delay, as in the case of the direct addition of GTP to FtsZ, could be due to two possibilities: (i). NDK (using the phosphate from ATP) generated GTP by directly phosphorylating the GDP remaining bound to the GDP-precharged FtsZ or (ii). NDK phosphorylated the free GDP, which might have got dissociated from the GDP-precharged FtsZ, to GTP, which in turn bound back to FtsZ, or got exchanged with the yet undissociated GDP on the FtsZ, triggering polymerisation. The binding constant (K_b_) values for GTP and GDP were reported to be 330 ± 80 μM and 110 ± 40 μM, respectively, for *Methanococcus jannaschii* FtsZ [60]. Although the binding constants for GTP and GDP are not known for mycobacterial FtsZ, exchange of GTP directly into the curved conformation of GDP-protofilaments has been reported [61]. Before finding out whether NDK directly phosphorylates the GDP on the FtsZ or the free GDP that got dissociated from the FtsZ, the generation of γ^32^P-GTP from GDP and γ^32^P-ATP by NDK was first confirmed.

**Fig 3:**
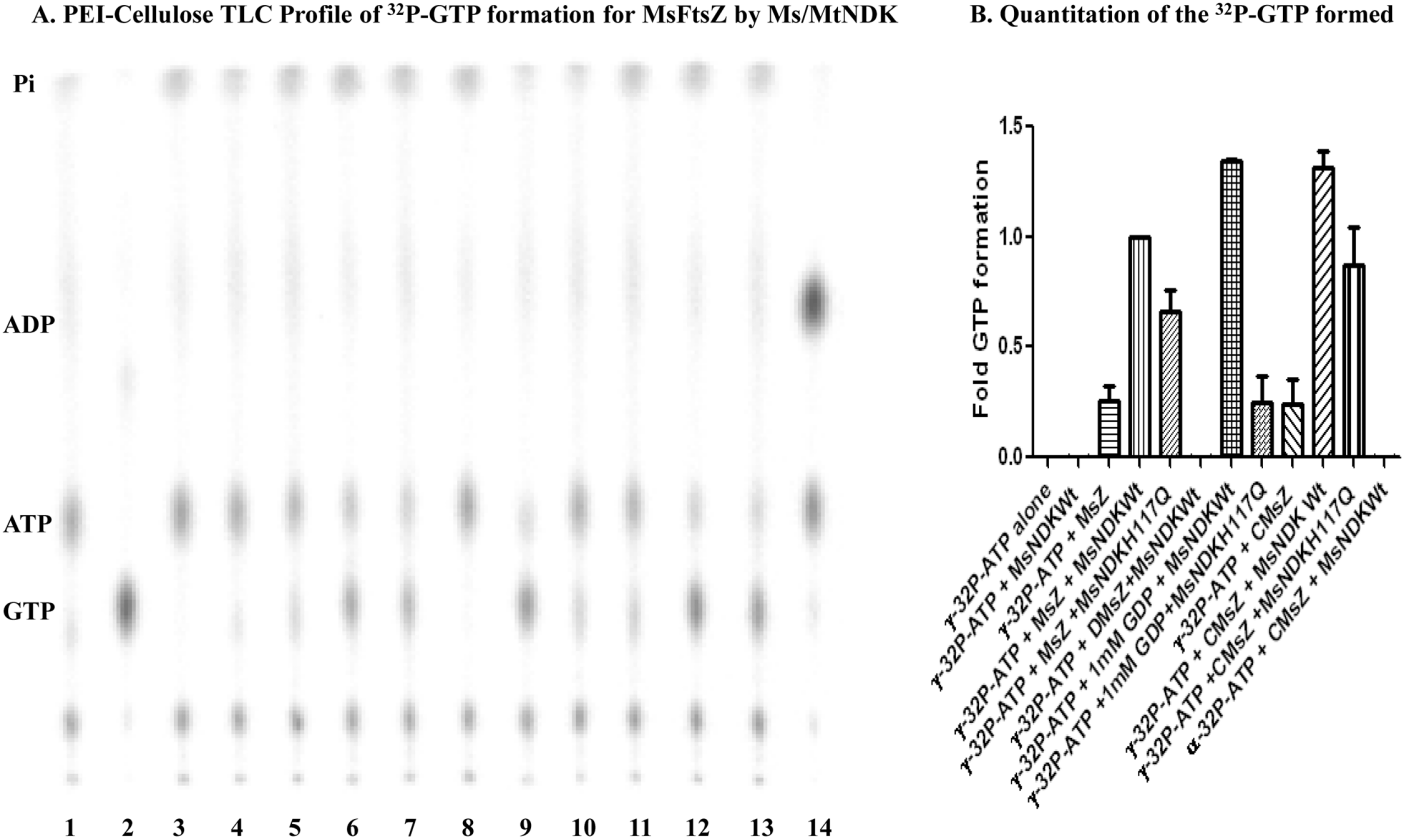
PEI-cellulose TLC profile of ^32^P-GTP formation during MsNDK-triggered MsFtsZ polymerisation and its quantitation. (**A**) Formation of GTP analysed on TLC. (**B**) Quantitation of the GTP formed. (**A**) The TLC profile of ^32^P-GTP formation. Lanes: 1. γ^32^P-ATP alone; 2. α^32^P-GTP alone; 3. γ^32^P-ATP + MsNDK-Wt; 4. γ^32^P-ATP + MsNDK-H117Q; 5. γ^32^P-ATP + Purified MsFtsZ; 6. γ^32^P-ATP + Purified MsFtsZ + MsNDK-Wt; 7. γ^32^P-ATP + Purified MsFtsZ + MsNDK-H117Q; 8. γ^32^P-ATP + GDP-predepleted MsFtsZ + MsNDK-Wt; 9. γ^32^P-ATP + GDP + MsNDK-Wt; 10. γ^32^P-ATP + GDP + MsNDK-H117Q; 11. γ^32^P-ATP + GDP-precharged MsFtsZ; 12. γ^32^P-ATP + GDP-precharged MsFtsZ + MsNDK-Wt; 13. γ^32^P-ATP + GDP-precharged MsFtsZ + MsNDK-H117Q; 14. α^32^P-ATP + GDP-precharged MsFtsZ + MsNDK-Wt. (**B**). Quantitation of the ^32^P-GTP from the TLC profile. The sample names are described on the X-axis. The samples, corresponding to the TLC lanes 2 and 4, where ^32^P-GTP formation is not expected, are not shown in the bar graph quantitation profile. CMsZ stands for GDP-charged MsFtsZ and DMsZ stands for GDP-depleted MsFtsZ.

The formation of γ^32^P-GTP, in the presence of MsNDK, from γ^32^P-ATP and the low levels of bound GDP on purified recombinant MsFtsZ and the GDP on the GDP-precharged MsFtsZ, was confirmed using thin layer chromatography (TLC). In the presence of MsNDK and purified MsFtsZ, low levels of γ^32^P-GTP formation could be observed (Fig 3, lane 6 in TLC, 4^th^ position in the bar graph). In the presence of MsNDK, increased γ^32^P-GTP formation was observed in the case of GDP-precharged MsFtsZ, as compared to that from the purified MsFtsZ (Fig 3, lane 12 in TLC, 10^th^ position in the bar graph; compare with lane 6 in TLC, 4^th^ position in the bar graph). It was quantitatively comparable to the formation of γ^32^P-GTP from free GDP by the transfer of the γ^32^Pi from γ^32^P-ATP to GDP by MsNDK (Fig 3, lane 9 in TLC, 7^th^ position in the bar graph). The mutant NDK showed negligible levels of γ^32^P-GTP formation (Fig 3, lane 10 in TLC, 8^th^ position in the bar graph).

The γ^32^P-GTP formation was not observed when GDP-predepleted MsFtsZ was used along with MsNDK and γ^32^P-ATP (Fig 3, lane 8 in TLC, 6^th^ position in the bar graph). Due to the partial activity of the mutant MsNDK-H117Q [13] (the negative control), some γ^32^P-GTP formation could be observed in the presence of the GDP-precharged MsFtsZ (Fig 3, lane 13 in TLC, 11^th^ position in the bar graph). Slightly less extent of γ^32^P-GTP formation was also observed when purified MsFtsZ (containing naturally bound GDP) was used with MsNDK-H117Q (Fig 3, lane 7 in TLC, 5^th^ position in the bar graph). Basal level of γ^32^P-GTP formation was observed, when the purified recombinant MsFtsZ alone was incubated with γ^32^P-ATP (Fig 3, lane 5 in TLC, 3^rd^ position in the bar graph). This could be due to the possibility that very low levels of *E*. *coli* NDK co-purify with the MsFtsZ from the MsFtsZ-overexpressed *E*. *coli* cells. Therefore, the naturally bound GDP on the purified MsFtsZ might be getting phosphorylated by the co-purifying *E*. *coli* NDK. Identical to the observations made in the case of MsFtsZ and MsNDK, γ^32^P transfer was found from γ^32^P-ATP to GDP in the presence of MtFtsZ and MtNDK (and its mutant as control) (S15 Fig; S1 Data). All these observations confirmed that NDK does phosphorylate GDP to GTP, using ATP as the phosphate donor, which in turn might be triggering FtsZ polymerisation.

### GDP Phosphorylation on GDP-FtsZ and Direct GTP Exchange for GDP on FtsZ

Before finding out whether NDK directly phosphorylates the GDP bound to FtsZ or the free GDP to GTP, which in turn is exchanged for the GDP on FtsZ, it was necessary to determine whether there exists any time difference between the direct exchange of GTP for the FtsZ-bound GDP and the generation of GTP from FtsZ-bound GDP by NDK. For this purpose, the ^32^P-GTP-FtsZ formed in the shortest practically monitorable time of 30 sec was immediately UV-crosslinked and quantitated. The formation of ^32^P-GTP-FtsZ was effected in two different ways: (i). 8.6 μM GDP-precharged MsFtsZ was incubated with α^32^P-GTP, for exchange with the GDP on the GDP-precharged MsFtsZ; (ii). 8.6 μM GDP-precharged MsFtsZ was incubated with γ^32^P-ATP and 0.1 μM NDK, wherein the γ^32^P-GTP-FtsZ formed by the NDK-mediated direct transfer of γ^32^P from γ^32^P-ATP to the GDP on the GDP-precharged MsFtsZ or by the phosphorylation of the GDP dissociated from the GDP-precharged MsFtsZ, might bind back FtsZ or exchange with the yet undissociated GDP. The assumption here is that the two modes of formation of ^32^P-GTP-FtsZ can be distinguished if one of the modes is slower than the other, with at least one of them taking more than 30 sec to generate γ^32^P-GTP-FtsZ.

**Fig 4:**
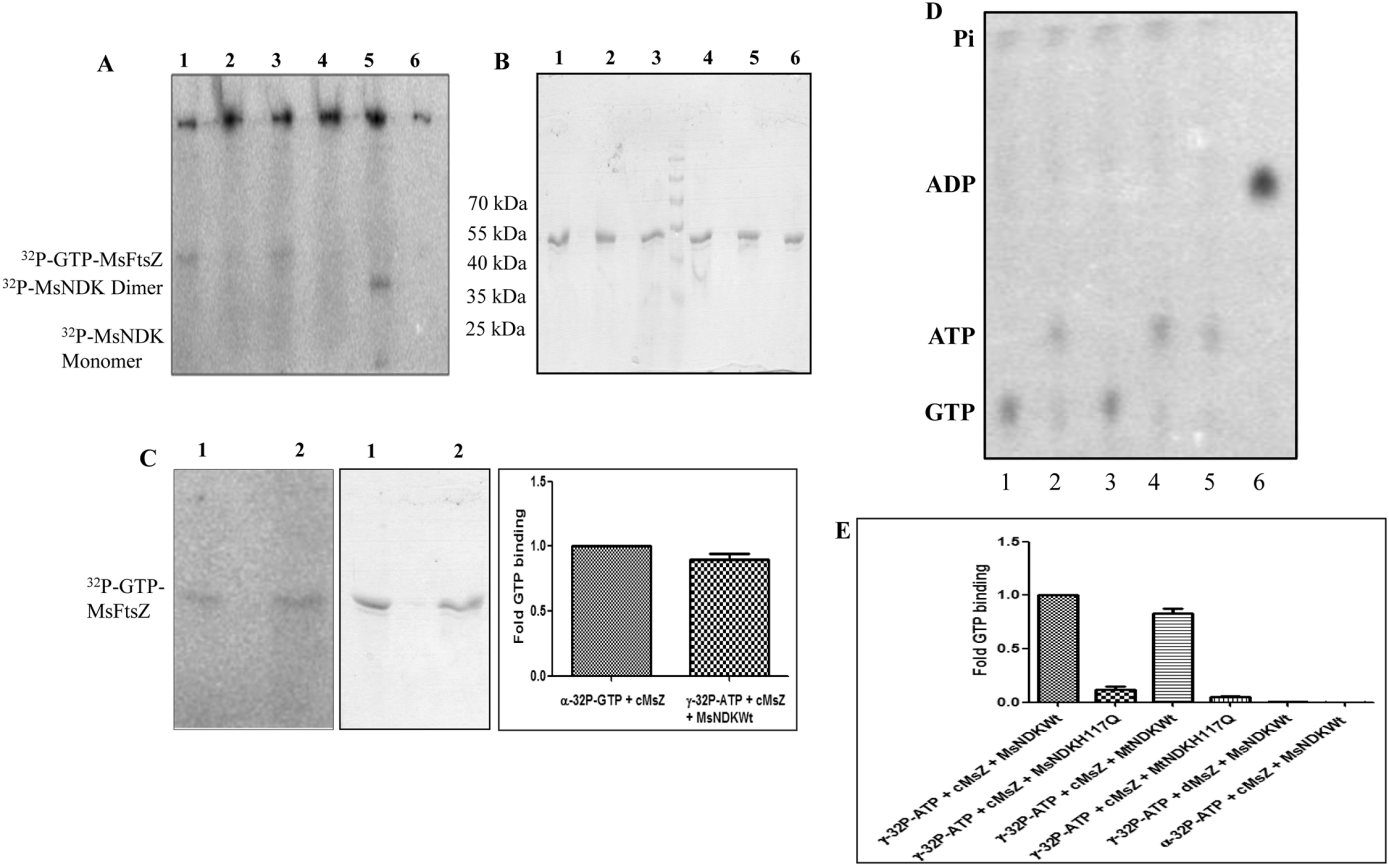
Assay for the binding of γ^32^P-GTP to MsFtsZ (30 sec), SDS-PAGE profile of the UV-crosslinked γ^32^P-GTP-MsFtsZ, and the quantitation of the γ^32^P-GTP-MsFtsZ formed. (**A**) SDS-PAGE profile of the UV-crosslinked γ^32^P-GTP-MsFtsZ bands and the autophosphorylated MsNDK monomer and dimer bands. Lanes: 1. GDP-precharged MsFtsZ + MsNDK-Wt + γ^32^P-ATP; 2. GDP-precharged MsZ + MsNDK-H117Q + γ^32^P-ATP; 3. GDP-precharged MsFtsZ + MtNDKWt + γ^32^P-ATP; 4. GDP-precharged MsFtsZ + MtNDK-H117Q + γ^32^P-ATP; 5. GDP-predepleted MsFtsZ + MsNDK-Wt + γ^32^P-ATP; see the autophosphorylated ^32^P-MsNDK dimer and monomer bands, due to the absence of GDP recipient substrate; 6. GDP-precharged MsFtsZ + MsNDK-Wt + α^32^P-ATP. (**B**) SDS-PAGE profile of the coomassie blue stained γ^32^P-GTP-MsFtsZ protein samples observed in (**A**). (**C**) Quantitation of the ^32^P-GTP-MsFtsZ formed from the UV-crosslinking. Left panel: phosphorimager profile of the UV-crosslinked ^32^P-GTP-MsFtsZ bands; Middle panel: the corresponding coomassie blue stained bands; Right panel: quantitation of the ^32^P-GTP-MsFtsZ. Lanes: 1. ^32^P-GTP-MsFtsZ formed from α^32^P-GTP and GDP-precharged MsFtsZ. 2. ^32^P-GTP-MsFtsZ formed from GDP-precharged MsFtsZ and γ^32^P-ATP, in the presence of MsNDK-wt. (**D**) PEI-cellulose TLC profile of γ^32^P-GTP formed. Lanes: 1. GDP-precharged MsFtsZ + MsNDK-Wt + γ^32^P-ATP; 2. GDP-precharged MsFtsZ + MsNDK-H117Q + γ^32^P-ATP; 3. GDP-precharged MsFtsZ + MtNDK-Wt + γ^32^P-ATP; 4. GDP-precharged MsFtsZ + MtNDK-H117Q + γ^32^P-ATP; 5. GDP-predepleted MsFtsZ + MsNDK-Wt + γ^32^P-ATP; 6. GDP-precharged MsFtsZ + MsNDK-Wt + α^32^P-ATP; 7. (**E**) Quantitation of the γ^32^P-GTP-MsFtsZ formed in the corresponding lanes 1–6 of panel A. cMsZ stands for GDP-charged MsFtsZ and dMsZ stands for GDP-depleted MsFtsZ.

The formation of γ^32^P-GTP from γ^32^P-ATP and GDP by MsNDK, and its binding to FtsZ, along with the formation of the autophosphorylated ^32^P-NDK reaction intermediate [13], were verified on the SDS-PAGE for the presence of UV-crosslinked γ^32^P-GTP-MsFtsZ formed from the 30 sec reaction (Fig 4A), and on the corresponding coomassie blue stained profile for the proteins (Fig 4B). Comparable extents of the presence of ^32^P-GTP were observed on γ^32^P-GTP-MsFtsZ within the 30 sec of the addition of: (i). α^32^P-GTP to GDP-precharged MsFtsZ and (ii). γ^32^P-ATP to GDP-precharged MsFtsZ in the presence of MsNDK (Fig 4C, bars 1 and 2 in the right panel and lanes 1 and 2 in the left and middle panels). When the sample (ii), without UV-crosslinking, was boiled and loaded onto TLC plate, the formation of γ^32^P-GTP could be noted within the 30 sec of incubation of GDP-precharged MsFtsZ and γ^32^P-ATP with MsNDK or MtNDK (Fig 4D, lanes 1 and 3 in the TLC, respectively). The MsNDK-H117Q and MtNDK-H117Q mutants could synthesise only negligible levels of γ^32^P-GTP from γ^32^P-ATP and GDP (Fig 4D, lanes 2 and 4 in the TLC, respectively). Consequentially, the extent of γ^32^P-GTP binding to MsFtsZ was also negligible (Fig 4E, bars 2 and 4 in the bar graph, respectively).

Substituting α^32^P-ATP for γ^32^P-ATP did not show any ^32^P-GTP binding to FtsZ (Fig 4E, position 6 in the bar graph). Concurrently, the formation of α^32^P-ADP, due to the transfer of unlabeled γ-phosphate of α^32^P-ATP to GDP, could be noted (Fig 4D, lane 6 in TLC). These observations confirmed that the GDP phosphorylation by NDK was mediated through the transfer of γ^32^P of γ^32^P-ATP to GDP to form γ^32^P-GTP. When GDP-depleted FtsZ was incubated with γ^32^P-ATP and MsNDK, in the absence of any free GDP, γ^32^P-GTP formation was not observed (Fig 4D, lane 5 in the TLC) and consequentially γ^32^P-GTP was not found on the MsFtsZ (Fig 4E, position 5 in the bar graph). In identical experiments using MtFtsZ and MtNDK and its mutant, MtNDK-H117Q, similar observations could be made for the γ^32^P-GTP binding to MtFtsZ and phosphorylation of GDP-precharged MtFtsZ by MtNDK by the γ^32^P transfer from γ^32^P-ATP to GDP (S16 Fig; S1 Data).

These observations showed that the formation of γ^32^P-GTP-FtsZ in both the following reactions occurred within 30 sec, which was the earliest practically feasible time at which γ^32^P-GTP-FtsZ formation could be detected; (i). direct phosphorylation of the GDP on the GDP-precharged FtsZ or from the GDP dissociated from the GDP-precharged FtsZ, which then bound back to FtsZ; (ii). direct FtsZ binding of γ^32^P-GTP that was formed from the GDP, on the GDP-precharged FtsZ, which got exchanged into the medium. Both these reactions occurred within 30 sec due to the fast kinetics of GTP binding to FtsZ, leading to polymerisation [41], and the high catalytic turnover rates of NDK [62,63]. Owing to these practical reasons, it was not possible to ascertain whether NDK generated γ^32^P-GTP-FtsZ by directly phosphorylating the GDP bound to FtsZ or by phosphorylating the GDP that got exchanged from FtsZ into the medium, which later bound back to FtsZ.

### NDK Phosphorylates the FtsZ-Bound GDP in the Absence of Free GDP

In view of the difficulty to distinguish between the two possibilities in 30 sec reaction time, attempts were made to find out whether NDK shows a preference for the phosphorylation of the GDP bound to FtsZ or for free GDP. This differential preference, if exists, may be indicated by a difference in the time taken for the formation of γ^32^P-GTP from the GDP bound to FtsZ and from the free GDP, in the presence of NDK and γ^32^P-ATP. For this purpose, two reactions of 30 sec duration (the shortest practically monitorable time) were set up: (i). GDP-precharged FtsZ was incubated with NDK and γ^32^P-ATP, in the absence of 1 mM exogenous GDP; (ii). GDP-predepleted FtsZ was incubated with exogenously supplied 1 mM GDP, NDK, and γ^32^P-ATP. Immediately after the 30 sec reactions, each sample was divided into two parts, one of which was boiled and loaded onto TLC sheet, developed, and exposed to phosphorimager to detect the γ^32^P-GTP formed, followed by quantitation of the spot on TLC. In the other part, the γ^32^P-GTP formed, on FtsZ itself or subsequently bound to FtsZ, was UV-crosslinked to FtsZ, fractionated on SDS-PAGE, and exposed to phosphorimager to detect γ^32^P-GTP-FtsZ.

**Fig 5:**
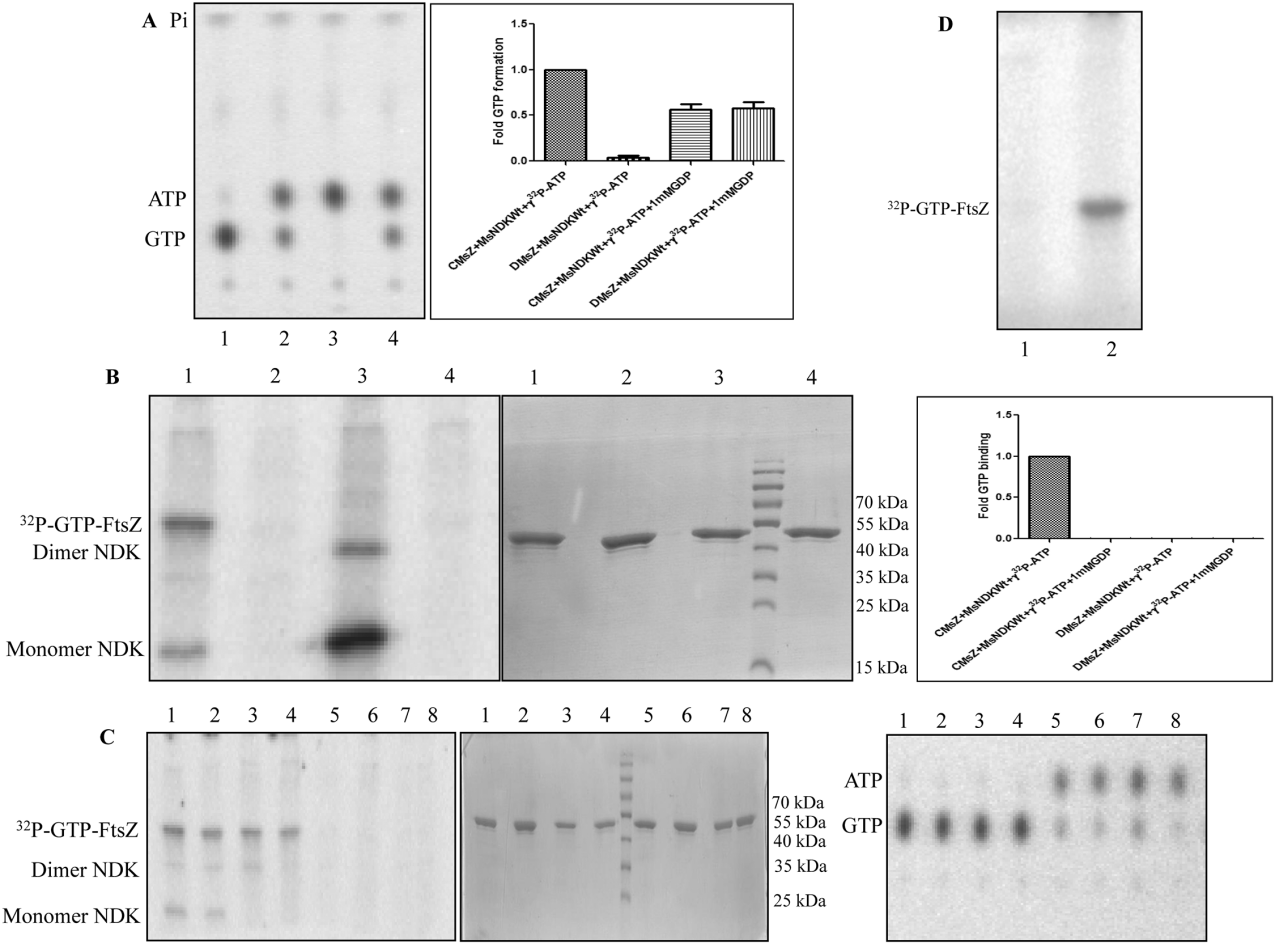
Assay for the formation of ^32^P-GTP from GDP bound to MsFtsZ and from free GDP. (**A**) **Left panel**: ^32^P-GTP formation assay (30 sec) by PEI-cellulose TLC. Lanes: 1. GDP-precharged MsFtsZ + MsNDK-Wt + γ^32^P-ATP; 2. GDP-precharged MsFtsZ + MsNDKWt + γ^32^P-ATP + GDP; 3. GDP-predepleted MsFtsZ + MsNDK-Wt + γ^32^P-ATP; 4. GDP-predepleted MsFtsZ + MsNDK-Wt + γ^32^P-ATP + GDP. **Right panel**: Quantitation of the ^32^P-GTP spots in the TLC profile, using phosphorimager. Description of the samples is given within the diagram. (**B**) Phosphorimager profile of the UV-crosslinked, SDS-PAGE fractionated γ^32^P-GTP-FtsZ samples (**left panel**), Coomassie blue stained protein profile of the corresponding γ^32^P-GTP-FtsZ samples (**middle panel**), and the quantitation of the corresponding γ^32^P-GTP-FtsZ samples (**right panel**). Lanes: 1. GDP-precharged MsFtsZ + MsNDK-Wt + γ^32^P-ATP; 2. GDP-precharged MsFtsZ + MsNDKWt + γ^32^P-ATP + GDP; 3. GDP-predepleted MsFtsZ + MsNDK-Wt + γ^32^P-ATP; 4. GDP-predepleted MsFtsZ + MsNDK-Wt + γ^32^P-ATP + GDP. (**C**) Phosphorimager profile of the SDS-PAGE fractionated UV-crosslinked γ^32^P-GTP-FtsZ (**left panel**), the coomassie blue stained profile of the corresponding γ^32^P-GTP-FtsZ protein samples (**middle panel**), and the TLC profile of the assay for the γ^32^P-GTP formed in the corresponding samples (**right panel**) for MtFtsZ or MsFtsZ. Lanes: 1. GDP-precharged MtFtsZ + MtNDK-Wt + γ^32^P-ATP; 2. Denatured-refolded GDP-recharged MtFtsZ + MtNDK-Wt + γ^32^P-ATP; 3. GDP-precharged MsFtsZ + MsNDK-Wt + γ^32^P-ATP; 4. Denatured-refolded GDP-recharged MsFtsZ + MsNDK-Wt + γ-^32^P-ATP; 5. GDP-precharged MtFtsZ + MtNDK-Wt + γ^32^P-ATP + GDP; 6. Denatured-refolded GDP-recharged MtFtsZ + MtNDK-Wt + γ^32^P-ATP + GDP; 7. GDP-precharged MsFtsZ + MsNDK-Wt + γ^32^P-ATP + GDP; 8. Denatured-refolded GDP-recharged MsFtsZ + MsNDK-Wt + γ^32^P-ATP + GDP. All the 8 samples were analysed after the 30 sec reaction mentioned in the text. (**D**) UV-crosslinking assay. Lanes: 1. GDP-cross-linked MsFtsZ + MsNDK-Wt + γ^32^P-ATP; 2. MsFtsZ + α^32^P-GTP (cross-linked post-binding).

TLC profile showed that γ^32^P-GTP was formed within 30 sec when GDP-precharged MsFtsZ was incubated with MsNDK and γ^32^P-ATP (in the absence of 1 mM exogenous GDP) (Fig 5A, lane 1, left panel). The γ^32^P-GTP formation within 30 sec could be observed also when GDP-predepleted MsFtsZ was incubated with exogenous 1 mM GDP, MsNDK, and γ^32^P-ATP (Fig 5A, lane 4, left panel). The extent of γ^32^P-GTP formed in the case of GDP-predepleted MsFtsZ (in the presence of 1 mM exogenous GDP) was only about 60% of the γ^32^P-GTP formed in the case of GDP-precharged MsFtsZ (in the absence of 1 mM exogenous GDP) (Fig 5A, compare bars 4 & 1). Interestingly, the amount of the γ^32^P-GTP formed when the GDP-precharged MsFtsZ was incubated with MsNDK and γ^32^P-ATP, in the presence of 1 mM exogenous GDP, was also only about 60% of the γ^32^P-GTP formed in the case of GDP-precharged MsFtsZ (in the absence of 1 mM exogenous GDP) (Fig 5A, compare lanes 2 & 4, and bars 3 & 4, respectively). The negative control, wherein the GDP-predepleted MsFtsZ was incubated with MsNDK and γ^32^P-ATP (in the absence of 1 mM GDP) did not show any γ^32^P-GTP formation (Fig 5A, lane 3, and bar 2).

Corroborating the TLC profile on the γ^32^P-GTP formation, the SDS-PAGE profile of γ^32^P-GTP-FtsZ formation showed the presence of γ^32^P-GTP on the GDP-precharged MsFtsZ, in the absence of exogenous GDP, but not in the presence of exogenous GDP (Fig 5B, lanes 1 & 2 in the left panel, and bars 1 & 2 in the bar graph). It showed that the γ^32^P-GTP formed from the exogenous GDP might not yet have got exchanged with the GDP bound to the GDP-precharged MsFtsZ in 30 sec, even though the GTP formed amounted to about 60% of the GTP formed in the case of GDP-precharged MsFtsZ in the absence of GDP (Fig 5A, compare bars 3 & 1). An alternate possibility could be that the small amount of γ^32^P-GTP formed from the exogenous GDP was not able to compete with the excess of exogenous GDP for binding to MsFtsZ. Similarly, γ^32^P-GTP was not found on the GDP-predepleted MsFtsZ (in the presence of 1 mM GDP) in 30 sec (Fig 5B, lane 4 in the left panel, and bar 4 in the bar graph). Again, it implied that the γ^32^P-GTP formed from the exogenous GDP might not yet have bound to the GDP-predepleted MsFtsZ in 30 sec, even though the γ^32^P-GTP formed amounted to about 60% of the GTP formed in the case of GDP-precharged MsFtsZ (in the absence of exogenous GDP). The autophosphorylated intermediate of NDK could be seen in the absence of GDP (Fig 5B, left panel, lane 1) but not in the presence of exogenous GDP (Fig 5B, left panel, lanes 2 & 4). It showed that the phosphate group was transferred through the formation of the high energy NDK-phosphate intermediate, as it occurs in the NDK reaction [4–8]. The negative control, where GDP-predepleted MsFtsZ was incubated in the presence of MsNDK and γ^32^P-ATP, did not show γ^32^P-GTP on MsFtsZ (Fig 5B, lane 3 in the left panel, and bar 3 in the bar graph).

It was possible that any structural alteration of FtsZ, during the denaturation and refolding of FtsZ for GDP depletion, could have been the reason for the lack of γ^32^P-GTP binding to GDP-predepleted FtsZ. However, the polymerisation of GDP-depleted-renatured MsFtsZ, in the presence of GTP, and in the presence of NDK, GDP, and ATP, albeit at slower rate by yielding polymers of lower mass, ruled out the possibility of the GDP-depleted-renatured MsFtsZ as non-functional (S17A and S17B Fig). Also, the presence of γ^32^P-GTP on the GDP-predepleted (denatured-refolded) MtFtsZ or MsFtsZ, when recharged with GDP and incubated with MtNDK or MsNDK and γ^32^P-ATP for 30 sec, followed by UV-crosslinking, ruled out this possibility (Fig 5C, left panel, lanes 2 and 4, respectively). Thus, the presence of γ^32^P-GTP could be observed on FtsZ within 30 sec even upon recharging of GDP-predepleted (denatured-refolded) FtsZ, like in the case of the GDP-precharged MtFtsZ or MsFtsZ, in the presence of MtNDK or MsNDK and γ^32^P-ATP (Fig 5C, left panel, lanes 1 and 3, respectively). In all these four reactions, γ^32^P-GTP formation could be observed on TLC (Fig 5C, right panel, lanes 1–4). However, when GDP was exogenously given, along with GDP-precharged MsFtsZ or MtFtsZ or denatured-refolded GDP-recharged MsFtsZ or MtFtsZ, in the presence of MsNDK or MtNDK and γ^32^P-ATP, the γ^32^P-GTP could not be found on the FtsZ (Fig 5C, left panel, lanes 5–8), as the γ^32^P-GTP formation on the FtsZ was significantly lesser upon TLC analysis (Fig 5C, right panel, lanes 5–8). Interestingly, MsNDK did not phosphorylate the GDP that was already UV-cross-linked to MsFtsZ (Fig 5D, lane 1). Only the α^32^P-GTP, which subsequently got bound to FtsZ, could be cross-linked (Fig 5D, lane 2). Identical and equivalent observations were made for the phosphorylation of GDP in the GDP-precharged or GDP-predepleted MtFtsZ by MtNDK, in the presence of γ^32^P-ATP, and the binding of γ^32^P-GTP to MtFtsZ (S18 Fig; S1 Data). These experiments showed that NDK readily phosphorylates: (i). the FtsZ-bound GDP in the absence of free GDP in solution; (ii). free GDP preferentially over FtsZ-bound GDP, in the presence of free GDP.

### Detection and Confirmation of NDK-FtsZ Interaction *In Vitro* and *Ex Vivo*

#### Ni^2+^-NTA agarose pull-down assay reveals MtNDK-MtFtsZ interaction

**Fig 6:**
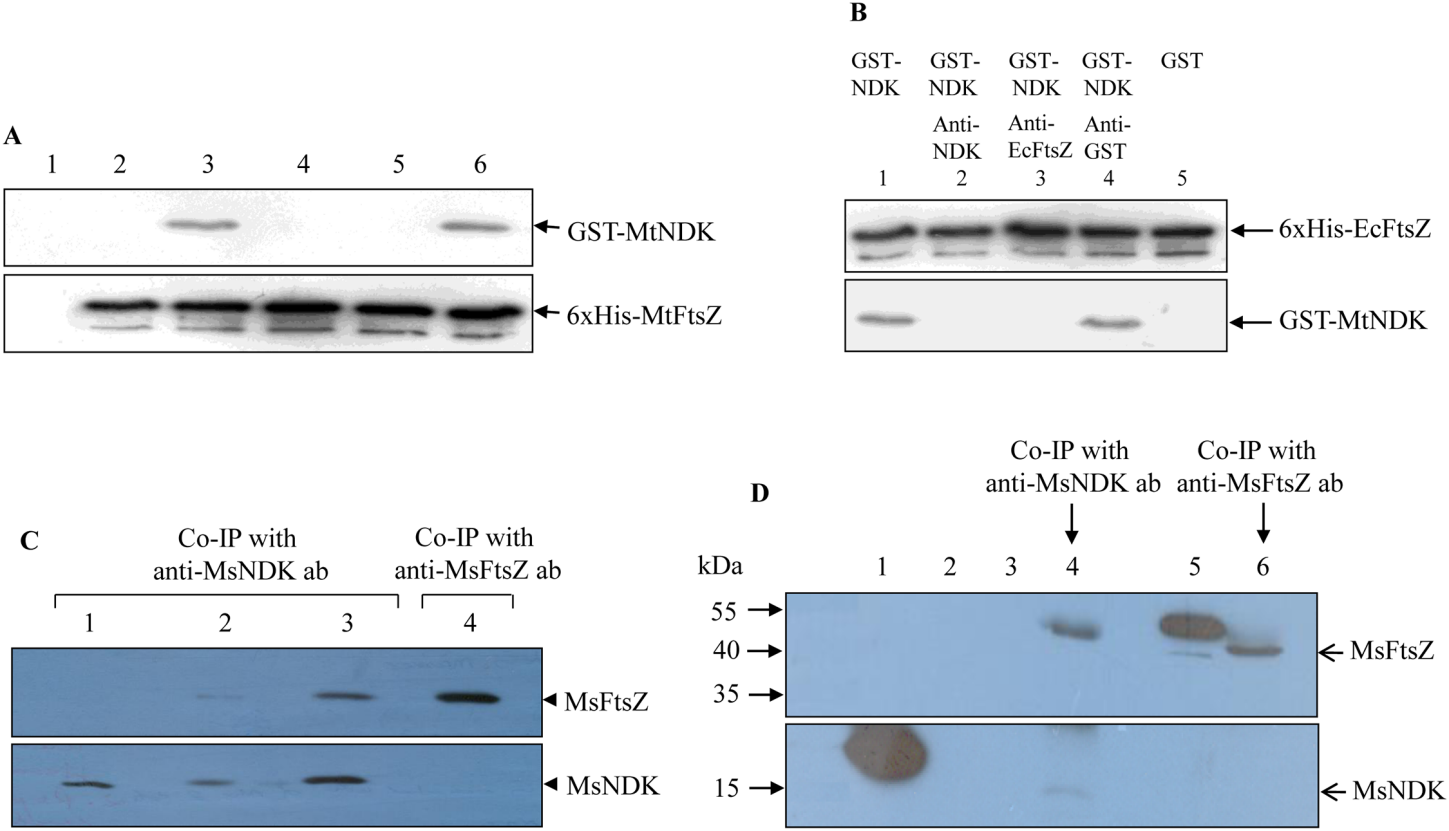
Pull-down assays and co-immunoprecipitation showing FtsZ-NDK interaction. (**A**) Pull-down assay using 6xHis-MtFtsZ and GST-MtNDK. Upper panel: GST-NDK is detected using anti-GST antibodies (mouse polyclonal). Lower panel: 6xHis-MtFtsZ is detected using anti-polyhistidine antibodies (mouse monoclonal). Lanes: 1. Ni^2+^-NTA agarose beads alone; 2. GST + 6xHis-MtFtsZ; 3. GST-MtNDK + 6xHis-MtFtsZ; 4. GST-MtNDK + 6xHis-MtFtsZ + anti-MtNDK antibodies (pre-incubated); 5. GST-MtNDK + 6xHis-MtFtsZ + anti-MtFtsZ antibodies (pre-incubated); 6. GST-MtNDK + 6xHis-MtFtsZ + anti-GST antibodies (pre-incubated). (**B**) Pull-down assay using 6xHis-EcFtsZ and GST-MtNDK. Upper panel: 6xHis-EcFtsZ is detected using anti-polyhistidine antibodies. Lower panel: GST-MtNDK is detected using anti-GST antibodies. Lanes: 1. GST-MtNDK + 6xHis-EcFtsZ; 2. GST-MtNDK + 6xHis-EcFtsZ + anti-MtNDK antibodies (pre-incubated); 3. GST-MtNDK + 6xHis-EcFtsZ + anti-EcFtsZ antibodies (pre-incubated); 4. GST-MtNDK + 6xHis-EcFtsZ + anti-GST antibodies (pre-incubated); 5. GST + 6xHis-EcFtsZ. Antibodies, whenever added to reaction mixtures were pre-incubated for 30 min at 4°C with the purified proteins at dilutions 1:100 for anti-MtNDK, anti-MtFtsZ, and anti-GST and 1:500 for anti-EcFtsZ before the addition of the cross-linker DTSP. The region where GST would have been on the blot is not shown. (**C**). Co-immunoprecipitation of recombinant MsNDK and MsFtsZ showing interaction. Lanes: 1–3. Co-IP with anti-MsNDK antibody; 4. Co-IP with anti-MsFtsZ antibody. Upper panel, western blotting with anti-MsFtsZ antibody; Lower panel, western blotting with anti-MsNDK antibody. Lanes: 1. MsNDK purified protein; 2. MsNDK, pre-incubated with anti-MsNDK antibody, followed by the addition of MsFtsZ; 3. MsNDK and MsFtsZ purified proteins; 4. MsFtsZ purified protein. (**D**) Co-immunoprecipitation of native MsNDK and MsFtsZ showing interaction in the total cell lysate. Lanes: 1. Purified recombinant 6x-His-MsNDK. 2. Protein A sepharose alone; 3. Protein A sepharose with *M*. *smegmatis* total cell lysate; 4. Co-IP with anti-MsNDK antibody; 5. Purified recombinant 6x-His-MsFtsZ; 6. Co-IP with anti-MsFtsZ antibody. Upper panel, western blotting with anti-MsFtsZ antibody; Lower panel, western blotting with anti-MsNDK antibody. Horizontal arrow heads indicates the endogenous MsNDK or MsFtsZ protein from the total cell lysate.

Phosphorylation of FtsZ-bound GDP raised the possibility of physical interaction between FtsZ and NDK. The interaction of MtNDK with MtFtsZ was first confirmed using Ni^2+^-NTA agarose pull-down assay. Affinity-purified 6xHis-MtFtsZ and GST-MtNDK were mixed in equimolar proportions in the presence of the cross-linker 3,3’-dithio-bis (N-hydroxysuccinimidyl propionate (DTSP). Ni^2+^-NTA agarose beads, which were used to pull-down 6xHis-MtFtsZ/GST-MtNDK complex, were washed free of unbound materials, the crosslink was reversed using 2-mercaptoethanol, and fractionated on SDS-PAGE. Immunoblotting with anti-GST antibodies revealed that GST-MtNDK got pulled down with 6xHis-MtFtsZ (Fig 6A, upper panel, lane 3). GST alone did not pull-down 6xHis-MtFtsZ (Fig 6A, upper panel, lane 2), showing that the interaction of GST-MtNDK with 6xHis-MtFtsZ was not due to the GST tag. Ni^2+^-NTA agarose beads alone also did not bind GST-MtNDK (Fig 6A, upper panel, lane 1), indicating that GST-MtNDK did not non-specifically interact with agarose beads. Pre-incubation of MtFtsZ-Ni^2+^-NTA agarose beads with anti-MtFtsZ antibody or pre-incubation of MtNDK with anti-MtNDK antibody, followed by western blotting with anti-GST antibody, showed abolition of the MtFtsZ-MtNDK interaction (Fig 6A, upper panel, lanes 4 and 5, respectively). On the other hand, anti-GST antibody did not abrogate the interaction (Fig 6A, upper panel, lane 6). Anti-polyhistidine monoclonal antibody detected the levels of 6xHis-MtFtsZ used for pull-down assay in all the samples (Fig 6A, lower panel, lanes 2–6). These observations showed that MtNDK does interact with MtFtsZ.

#### Ni^2+^-NTA agarose pull-down assay reveals MtNDK-EcFtsZ interaction

Since both the molecules, NDK and FtsZ, are conserved across bacterial genera [9–11,64], we further examined whether the interaction observed between MtFtsZ and MtNDK of mycobacterial system occurs between FtsZ of *E*. *coli* (EcFtsZ) and MtNDK as well. Therefore, in an identical approach under identical experimental conditions, with Ni^2+^-NTA agarose pull down experiments using GST-MtNDK and 6xHis-EcFtsZ in the presence of the cross-linker, DTSP, GST-MtNDK was found to interact with 6xHis-EcFtsZ (Fig 6B, lower panel, lane 1). Preincubation with anti-MtNDK or anti-EcFtsZ antibody blocked the interaction between MtNDK and EcFtsZ proteins (Fig 6B, lower panel, lanes 2 and 3, respectively), whereas anti-GST antibodies did not abolish the interaction (Fig 6B, lower panel, lane 4). These results proved that MtNDK does interact with EcFtsZ as well *in vitro*, revealing the conserved nature of the interaction between NDK and FtsZ, commensurate with the conserved structure of the proteins.

#### Co-immunoprecipitation *in vitro* confirms MsNDK-MsFtsZ interaction

In order to confirm NDK-FtsZ interaction, MsNDK was co-immunoprecipitated *in vitro* from a mixture of recombinant MsNDK and MsFtsZ proteins, using affinity-purified anti-MsNDK antibody. Western blotting of the co-immunoprecipitate for MsFtsZ and MsNDK independently showed the presence of both the proteins (Fig 6C, lane 3, of the top and bottom panels, respectively). However, reciprocal co-immunoprecipitation with anti-MsFtsZ antibody, and western blotting for MsFtsZ and MsNDK, showed only MsFtsZ but not MsNDK (Fig 6C, lane 4, of the top and bottom panels, respectively). Pre-incubation of MsNDK with anti-MsNDK antibody, prior to the addition of MsFtsZ and subsequent co-immunoprecipitation with anti-MsNDK antibody, showed reduced levels of MsFtsZ (Fig 6C, lane 2, top panel). Affinity purified anti-MsNDK antibody did not crossreact with either MsFtsZ or other proteins in the lysate (data not shown). These observations established that MsNDK does interact with MsFtsZ, like in the case of MtNDK-MtFtsZ interaction found using affinity blotting and pulldown assays.

#### Co-immunoprecipitation *ex vivo* confirms MsNDK-MsFtsZ interaction

Interaction between the native NDK and FtsZ was examined *ex vivo* in the *M*. *smegmatis* whole cell lysate. Co-immunoprecipitation of the lysate with anti-MsNDK antibody and western blotting with anti-MsFtsZ antibody showed the presence of MsFtsZ (Fig 6D, upper panel, lane 4). Western blotting of the co-immunoprecipitate with anti-MsNDK antibody showed the presence of MsNDK (Fig 6D, lower panel, lane 4). However, the reciprocal co-immunoprecipitation of the lysate using anti-MsFtsZ antibody and western blotting with anti-MsNDK antibody did not detect MsNDK band (Fig 6D, lower panel, lane 6), like in the case of co-immunoprecipitation with purified recombinant proteins using anti-MsFtsZ antibody (see Fig 6C, lane 4). The control sample, where the whole cell lysate was mixed with protein A-sepharose alone, did not detect any protein band, indicating the absence of any non-specific interaction of the proteins with protein A-sepharose (Fig 6D, upper panel lane 3). Taken together, different types of biochemical methods using FtsZ and NDK proteins from different mycobacterial species and other bacterial genus confirmed that the NDK-FtsZ interaction is conserved across bacterial species and genera.

## Discussion

### NDK Triggers FtsZ Polymerisation—A Novel Direct Role for NDK in FtsZ Function

The present study shows for the first time that the NDK of one mycobacterial species physically interacts with the FtsZ of the same mycobacterial species or of another mycobacterial species, to trigger FtsZ polymerisation by converting FtsZ-bound GDP or free GDP to GTP, using ATP. With this novel role of NDK in FtsZ polymerisation, which implies its influence on bacterial cytoskeleton and cell division, FtsZ becomes the new addition to the already known wide repertoire of protein factors with which NDK interacts to influence diverse metabolic functions in bacterial cells (reviewed in [65]; see [66–69] also). Conversely, considering the vast repertory of diverse proteins with which FtsZ interacts during cell division [70], NDK becomes the novel addition. The cross-species interaction between NDK and FtsZ, leading to FtsZ polymerisation, and the interaction of MtNDK with EcFtsZ, implied that the interaction and the consequential FtsZ polymerisation reaction are conserved across diverse bacterial genera. The conservation in the FtsZ-NDK interaction is in concurrence with the structural conservation of both NDK and FtsZ on their own accord across diverse bacterial genera [9–11,64].

### Conservation of the NDK-FtsZ Interaction in NDPK-Tubulin Interaction

Considering the fact that FtsZ is a bacterial cytoskeletal protein, the structure of which is conserved in eukaryotic tubulin [36,37], the NDK-FtsZ interaction has been found conserved in the NDPK-tubulin interaction as well. The association of NDPK with the tubulin purified from the brain cell lysates of guinea pig [31–33], chicken [34], and bovine [35], and NDPK assisting tubulin polymerisation by converting tubulin-bound GDP to GTP in the presence of nucleotides *in vitro* [31–33] stand testimony to the conservation of FtsZ-NDK interaction. Further instances include the activation of G proteins by NDK/NDPK by phosphorylating the G-protein bound GDP to GTP [18–25] and the activation of the molecular motor protein, dynamin, through the conversion of GDP to GTP [29]. In addition, NDKs/NDPKs function as the source of GTP for the elongation factor EF-Tu for protein biosynthesis [27], for dynamin for synaptic vesicle recycling [28] and for membrane remodeling [29]. NDPK supplies GTP also for diverse cytoskeletal components and regulators, such as actin-binding proteins, intermediate filaments, and cytoskeletal attachment structures (adherens junctions, desmosomes, and focal adhesions) [reviewed in 30]. In all these interactions, the NDK (NDPK) assists activation of specific GTP-requiring proteins, through the conversion of free or protein-bound GDP to GTP, using ATP (or other NTPs) as the phosphate donor. These instances show the highest degree of conservation of the interaction between NDK(NDPK) and cytoskeletal proteins. It indicates the wide range of influence that NDK (NDPK) wields on diverse cellular processes.

### NDK-FtsZ Interaction Is Robust

Technically different approaches (pulldown assay and co-immunoprecipitation of recombinant and native proteins) showed interaction between FtsZ and NDK from different bacterial species and genera *in vitro* and *ex vivo*. This revealed the robustness of the interaction. Co-immunoprecipitation using anti-NDK antibody and western blotting with anti-FtsZ antibody could detect FtsZ. However, the reciprocal co-immunoprecipitation using anti-MsFtsZ antibody and western blotting with anti-MsNDK antibody did not detect MsNDK. This may be due to the possibility of MsFtsZ antibody interacting with the region on MsFtsZ recognised by MsNDK. The pulldown assays showed interaction between NDK and FtsZ only in the presence of the cross-linker (DTSP). The inability to pulldown these proteins in the absence of the cross-linker might be due to the incompatibility of the fusion of the dimeric GST with the hexameric MtNDK [15]. In fact, the detectable interaction between NDK and FtsZ in their native form lacking any tag *ex vivo* (in the cell lysate) or in their short-sized 6xHis-tagged form *in vitro*, supports this possibility.

### Non-Specificity in the Use of NTP in the NDK-FtsZ Interaction

In the interaction of NDK with FtsZ to trigger FtsZ polymerisation, the NDK has retained its characteristic substrate non-specificity in using all the four NTPs as the phosphate donor [5]. The NDK-triggered FtsZ polymerisation, in the presence of ATP, is as efficient as the conventional mode of polymerisation of FtsZ through the direct binding of GTP to FtsZ. The partial support of FtsZ polymerisation by the mutant NDK is in concurrence with the report that the active site mutation does not completely abolish activity owing to contribution by several other residues [12,13]. The mycobacterial NDK using NTPs other than ATP also for FtsZ polymerisation is similar to the eukaryotic NDPK utilizing UTP and CTP also as the phosphate donor for the generation of GTP to assist tubulin polymerisation [31]. It was interesting to note that for the polymerisation of MtFtsZ, MsNDK and MtNDK could use only CTP and TTP, but not UTP, although UTP could be utilised by the same NDKs to trigger the polymerisation of MsFtsZ. Even the TTP-utilised polymerisation reaction was 80 sec delayed, compared to the usual 40 sec delay in the reaction utilising CTP. These variations could probably be due to the possible structural difference(s) in the interaction of the NDKs with the two FtsZ molecules. A three-dimensional comparative structural analysis of the NDK-MtFtsZ and NDK-MsFtsZ complexes is required to explain the variations.

### Direct Phosphorylation of FtsZ-Bound GDP by NDK

Instant FtsZ polymerisation of the GDP-precharged FtsZ within 40 sec post addition of ATP in the presence of NDK, similar to FtsZ polymerisation upon the direct addition of GTP, might be due to all of the FtsZ protein molecules being loaded with GDP. The 40 sec, kept as the time interval for the polymerisation data acquisition in the 90° light scattering assay, was the minimum time needed for the manual addition and mixing of ATP or GTP. From GDP and ATP, the NDK-mediated synthesis of GTP needs to reach the threshold concentration of GTP for FtsZ polymerisation [41,42,52], to bind FtsZ to trigger polymerisation. Thus, the more than 40 sec delay in FtsZ polymerisation in the presence of GDP, ATP, and NDK, might be due to the time taken for the build-up of GTP concentration to the required threshold levels for FtsZ polymerisation. The absence of the 40 sec delay in FtsZ polymerisation, when GDP-precharged FtsZ was exposed to ATP in the presence of NDK, strengthens this possibility. Further, the difference in the time for the NDK-triggered polymerisation of GDP-precharged and GDP-not-precharged FtsZ may be indicative of the possibility of the direct phosphorylation of the FtsZ-bound GDP by NDK, which occurred within 30 sec. However, the possibility of GDP coming off from the GDP-precharged FtsZ into the medium, getting phosphorylated by NDK and ATP, and binding back to the FtsZ, could not be ruled out due to this reaction also occurring within 30 sec. Since the incorporation of GTP into *E*. *coli* FtsZ has been found to occur within 10 sec [71] and the phosphoryl transfer reaction of NDK is too fast to be measured even using stopped-flow methods [62], it was not possible to distinguish between the two possibilities any further *in vitro*, and much less *in vivo*. Nevertheless, the physical association between the two proteins may be another indication of the direct phosphorylation of the FtsZ-bound GDP by NDK.

At this juncture, it is interesting to note that NDK phosphorylated free GDP, in preference to FtsZ-bound GDP, although only to 60%. However, the phosphorylation of FtsZ-bound GDP occurred readily and completely within 30 sec, when free GDP was absent. Similar behavior was observed with NDPK and tubulin, wherein more than 75% of the GTP formed was from the GDP bound to tubulin and only 10% of the exogenously added GTP bound to tubulin [31]. Therefore, although free GDP might be competing with the GDP bound to MsFtsZ, the phosphotransfer activity of NDK might be faster on the GDP bound to FtsZ, compared to the free GDP in solution. Further, the free GTP that was formed by the direct phosphorylation of free GDP by NDK did not also bind FtsZ within 30 sec. A similar situation has been reported in the case of studies using pig brain tubulin and NDPK [31,33]. These studies have shown that the catalytic effect of the NDPK is faster than the rate of nucleotide exchange on tubulin at the E site (GTP exchangeable site) and therefore tubulin-GDP complex itself is a substrate for NDPK [31,33]. It strengthens the possibility of the direct phosphorylation of tubulin-bound GDP by NDPK. These observations on the interaction between NDPK and tubulin, which are the structural and functional homologues of NDK and FtsZ [9–11,36,37,64], are strongly in favour of the direct phosphorylation of FtsZ-bound GDP by NDK.

Mammalian NDPK phosphorylates the tubulin-bound GDP, which is not cross-linked [31–33]. However, it is not known whether it will phosphorylate the bound GDP that is UV-cross-linked to tubulin. Interestingly, human NDPK (NM23) could phosphorylate the GDP that was UV-cross-linked to the Rad protein, a Ras-related GTPase [23]. On the contrary, although MsNDK could phosphorylate the GDP bound to FtsZ, it did not phosphorylate the FtsZ-bound GDP that was UV-cross-linked to FtsZ. There are specific primary structural differences between MsNDK and human NDPK (NM23), although both are overall hexameric in nature [14,15,72]. Probably, these differences between MsNDK and NM23, and the overall structural difference between FtsZ and Rad proteins, might have contributed to the difference in the accessibility of the UV-cross-linked GDP bound to FtsZ and Rad to MsNDK and NM23, respectively.

### The Possible Biological Context of NDK-Triggered FtsZ Polymerisation in the Cell

FtsZ polymerisation is sensitive to the GTP/GDP ratio [73] and the ratio of GTP/GDP is high in *Salmonella typhimurium* and *Escherichia coli* [74,75]. The FtsZ concentrations are also high (in μM range) in many bacterial cells [76–80], including *M*. *tuberculosis* and *M*. *smegmatis* [81]. Thus, the intracellular concentrations of FtsZ and GTP are sufficient for FtsZ polymerisation under normal growth conditions [82]. Similarly, the NDK-triggered FtsZ polymerisation will be dependent on the intracellular concentration of NDK and the binding affinity of the two proteins. The physiological concentration of NDK in *M*. *smegmatis* was found to be 0.13 μM by western blotting [83]. Thus, the concentrations of NDK (0.10 μM) and FtsZ (8.6 μM) used in our *in vitro* experiments were not limiting, but well comparable to the physiological concentrations of the two proteins. Nevertheless, the presence of MsFtsZ-MsNDK complex in the mid-log phase cell lysates (see Fig 6) showed that the NDK-FtsZ complex is naturally existent in the cells. These facts raise the question as to when the NDK-mediated FtsZ polymerisation is required in the life cycle of the cell.

FtsZ polymerisation has been found to involve continuous exchange of FtsZ subunits for fresh rounds of polymerisation [61,78,84,85], which involves continuous GTP hydrolysis as well. However, the source for the continuous supply of GTP remains unknown. It is possible that the GDP formed due to the GTP hydrolysis during FtsZ polymerisation can be quickly converted into GTP by the NDK. From this standpoint, NDK may be involved in the FtsZ polymerisation during active cell division process. Another context for the NDK-mediated GDP-to-GTP conversion for FtsZ polymerisation may be the GTP limiting conditions in the cell. For instance, a decrease in the guanine nucleotide pool has been observed as the cells enter stationary phase [86–88]. FtsZ being a cytokinetic as well as a cytoskeletal protein [36,37], polymerised FtsZ is required to meet the low levels of cell division and/or cytoskeletal maintenance under such GTP-limiting conditions as well. Under such conditions, NDK can tilt the ratio of GDP-FtsZ to GTP-FtsZ in the cell to enable FtsZ polymerisation. Thus, the NDK-mediated polymerisation of FtsZ, which is as efficient as the polymerisation by the direct binding of GTP, may have relevance during the actively growing phase of the cell as well as under GTP-limiting conditions in the cell.

## Supporting Information

S1 Data Supplementary Data. (DOCX)

S1 Fig Polymerisation of MsFtsZ at different concentrations of Mg^2+^. (TIF)

S2 Fig Transmission electron micrographs of polymerised samples of MsFtsZ. (TIF)

S3 Fig Polymerisation profiles by 90° Light Scattering (LS) assay of MtFtsZ. (TIF)

S4 Fig Transmission electron micrographs of polymerised samples of MtFtsZ. (TIF)

S5 Fig SDS-PAGE profile of the FtsZ polymer pelleting assay for MtFtsZ polymerisation and its quantitation. (TIF)

S6 Fig 90° Light Scattering (LS) assay profiles of MsFtsZ polymerisation in the presence of CTP. (TIF)

S7 Fig 90° Light Scattering (LS) assay profiles of MsFtsZ polymerisation in the presence of TTP. (TIF)

S8 Fig 90° Light Scattering (LS) assay profiles of MsFtsZ polymerisation in the presence of UTP. (TIF)

S9 Fig 90° Light Scattering (LS) assay profiles of GDP-precharged MsFtsZ polymerisation in the presence of CTP. (TIF)

S10 Fig 90° Light Scattering (LS) assay profiles of GDP-precharged MsFtsZ polymerisation in the presence of TTP. (TIF)

S11 Fig 90° Light Scattering (LS) assay profiles of GDP-precharged MsFtsZ polymerisation in the presence of UTP. (TIF)

S12 Fig 90° Light Scattering (LS) assay profiles of MtFtsZ polymerisation in the presence of CTP. (TIF)

S13 Fig 90° Light Scattering (LS) assay profiles of MtFtsZ polymerisation in the presence of TTP. (TIF)

S14 Fig 90° Light Scattering (LS) assay profiles of MtFtsZ polymerisation in the presence of UTP. (TIF)

S15 Fig PEI-cellulose TLC profile of ^32^P-GTP formation during MtNDK-triggered MtFtsZ polymerisation and its quantitation. (TIF)

S16 Fig Assay for the binding of γ^32^P-GTP to MtFtsZ (30 sec), SDS-PAGE profile of the UV-crosslinked γ^32^P-GTP-MtFtsZ, and the quantitation of the γ^32^P-GTP-MtFtsZ formed. (TIF)

S17 Fig Polymerisation potential of GDP-depleted and renatured MsFtsZ. (TIF)

S18 Fig Assay for the formation of ^32^P-GTP from GDP bound to MtFtsZ and from free GDP. (TIF)

S1 Table List of bacterial strains. (DOCX)

S2 Table List of oligonucleotide primers. (DOCX)

S3 Table List of the plasmid vectors. (DOCX)

S1 Text Supplementary Text. (DOCX)
