# Supplementary material for: NDK Interacts with FtsZ and Converts GDP to GTP to Trigger FtsZ Polymerisation - A Novel Role for NDK
Source: PLoS One. 2015 Dec 2;10(12):e0143677. doi: 10.1371/journal.pone.0143677 (PMC4668074; doi:10.1371/journal.pone.0143677)
Supplement: S1 Data — (DOCX) [file pone.0143677.s001.docx]

**S1 Data (Supplementary Data)**

**NDK Interacts with FtsZ and Converts GDP to GTP to Trigger FtsZ polymerisation –**

**A Novel Role for NDK**

Saurabh Mishra^1^, Kishor Jakkala^1^, Ramanujam Srinivasan^1,#a^, Muthu Arumugam^1,#b^, Raghavendra Ranjeri^1^, Prabuddha Gupta^1,#c^, Haryadi Rajeswari^1^, and Parthasarathi Ajitkumar^1,^*

MsNDK and MtNDK facilitate MtFtsZ polymerisation – identified using 90° LS Assay

Like in the case of MsFtsZ polymerisation, MtNDK and MsNDK facilitated polymerisation of MtFtsZ also. When incubated with 0.1 µM of MtNDK, 1 mM GDP, and 1 mM ATP, purified 8.6 µM MtFtsZ showed significant extent of polymerisation, but with a delay of about 40 sec post-addition of ATP, as revealed by the 90° light scattering (LS) assay (S3 Fig, ■ and × symbols, respectively). Strangely, unlike in the case of MsFtsZ, where the mutant NDKs supported polymerisation albeit at a slower rate (Fig 1A), the MtNDK-H117Q and MsNDK-H117Q (negative control) could not support MtFtsZ polymerisation (S3 Fig, ▲ and ж symbols, respectively). The mutant NDKs being the same, one possibility for this difference may be the difference in the structural features between MsFtsZ and MtFtsZ that govern their interaction with NDKs, which merits further investigation. As expected, the positive control (with 1 mM GTP) showed instant polymerisation without the 40 sec delay (S3 Fig, ♦ symbol). The negative controls, without GDP in the presence of NDK, MtFtsZ, and ATP (S3 Fig, ־ and + symbols, respectively), and without MtFtsZ in the presence of NDK, GDP, and ATP (S3 Fig, ○ and — symbols, respectively), did not show polymerisation. It is possible that the proportion of GDP-bound MtFtsZ may be low and therefore not sufficient enough to trigger polymerisation without the addition of exogenous GDP. Transmission electron micrographs of the MtFtsZ polymers showed that the MtFtsZ polymers formed through MtNDK facilitated polymerisation (using ATP and GDP) were morphologically identical to those formed by the direct addition of GTP to MtFtsZ, in the absence of MtNDK (S4 Fig). These observations showed that MsNDK and MtNDK can trigger MtFtsZ polymerisation in the presence of GDP and ATP.

MtFtsZ polymer pelleting assay confirms MtNDK- and MsNDK-facilitated MtFtsZ polymerisation

MtFtsZ polymerisation by MsNDK and MtNDK, in the presence of GDP and ATP, was confirmed using MtFtsZ polymer pelleting assay, wherein the pellet contained polymerised MtFtsZ (S5A Fig, lanes 1 and 3, respectively), while the unpolymerised MtFtsZ was present in the supernatant (S5B Fig, 11 and 13, respectively). The extent of MtFtsZ present as polymer in the pellet fraction in the experimental samples was comparable to that in the case of MtFtsZ polymer formed upon the direct addition of GTP (S5A Fig, compare lanes 1 and 3, respectively, with lane 10).

MtNDK-H117Q and MsNDK-H117Q did not facilitate MtFtsZ polymerisation, in the presence of GDP and ATP, as revealed by the lower levels of MtFtsZ protein in the pellet, compared to that in the supernatant (S5 Fig, compare lanes 2 and 4 in the pellet fraction A, with lanes 12 and 14 in the supernatant fraction B, respectively). Neither MtNDK nor MsNDK facilitated MtFtsZ polymerisation in the absence of exogenously added GDP, as revealed by lower levels of MtFtsZ in the pellet fraction (S5A Fig, lanes 5 and 7 in the pellet fraction), compared to that in the supernatant fraction (S5B Fig, lanes 15 and 17 in the supernatant fraction). In the absence of MtFtsZ, neither the pellet nor the supernatant fraction contained any protein (S5 Fig, compare lanes 8 and 9 in the pellet fraction A, with lanes 18 and 19 in the supernatant fraction B, respectively). The respective quantitation is provided in S5C Fig.

MsNDK & MtNDK trigger MtFtsZ polymerisation using other NTPs

A notable difference in the case of MtFtsZ polymerisation triggered by MsNDK and MtNDK, using other NTPs, was that the NDKs could use only CTP and TTP, but not UTP, for the polymerisation of MtFtsZ (compare S12 and S13 Figs for CTP and TTP, respectively, with S14 Fig for UTP). Moreover, the TTP-utilised polymerisation reaction was delayed by 80 sec, as compared to the usual 40 sec delay in the reaction utilising ATP or CTP. The reaction using UTP showed only very negligible level of MtFtsZ and MsFtsZ polymerisation, which itself was delayed by 120 sec (S14 Fig). Thus, irrespective of the NTP used (except UTP), in the presence of exogenous GDP, NDK triggered polymerisation of recombinant MtFtsZ.

MtNDK converts GDP to GTP to trigger MtFtsZ polymerisation

The formation of γ^32^P-GTP, in the presence of MtNDK, from γ^32^P-ATP and the low levels of bound GDP on purified recombinant MtFtsZ and the GDP on the GDP-precharged MtFtsZ, was confirmed using thin layer chromatography (TLC) on PEI-cellulose sheets. In the presence of MtNDK and purified MtFtsZ, low levels of γ^32^P-GTP formation could be observed (S15 Fig, lane 6 in TLC, 4^th^ position in the bar graph). In the presence of MtNDK, increased γ^32^P-GTP formation was observed in the case of the GDP-precharged MtFtsZ, as compared to that from the purified MtFtsZ (S15 Fig, lane 12 in TLC, 10^th^ position in the bar graph; compare with lane 6 in TLC, 4^th^ position in the bar graph). It was quantitatively comparable to the formation of γ^32^P-GTP from free GDP by the transfer of the γ^32^Pi from γ^32^P-ATP to GDP by MtNDK (S15 Fig, lane 9 in TLC, 7^th^ position in the bar graph). The mutant NDK showed negligible levels of γ^32^P-GTP formation (S15 Fig, lane 10 in TLC, 8^th^ position in the bar graph).

The γ^32^P-GTP formation was not observed when GDP-predepleted MtFtsZ was used along with MtNDK and γ^32^P-ATP (S15 Fig, lane 8 in TLC, 6^th^ position in the bar graph). Due to the partial activity of the mutant MtNDK-H117Q (the negative control), some γ^32^P-GTP formation could be observed in the presence of the GDP-precharged MtFtsZ (S15 Fig, lane 13 in TLC, 11^th^ position in the bar graph). Slightly less extent of γ^32^P-GTP formation was also observed when purified MtFtsZ (containing naturally bound GDP) was used with MtNDK-H117Q (S15 Fig, lane 7 in TLC, 5^th^ position in the bar graph). Negligible level of γ^32^P-GTP formation was observed, when the purified recombinant MtFtsZ alone was incubated with γ^32^P-ATP (S15 Fig, lane 5 in TLC, 3^rd^ position in the bar graph). This could be due to the possibility that low levels of *E. coli* NDK might be co-purifying with the MtFtsZ purified from the MtFtsZ-overexpressed *E. coli* cells. Therefore, due to the NDK-FtsZ conserved interaction (data shown in the main text), in which the naturally bound GDP on the purified MtFtsZ might be getting phosphorylated by the co-purifying *E. coli* NDK.

Direct GTP exchange for GDP on FtsZ and GDP phosphorylation on GDP-FtsZ

Having confirmed that NDK does convert GDP to GTP, using ATP, for FtsZ polymerisation, and before finding out whether NDK phosphorylates the GDP bound to FtsZ or the free GDP to GTP, which in turn is exchanged for the GDP on FtsZ, it was necessary to determine whether there exists any time difference between the direct exchange of GTP for the FtsZ-bound GDP and the generation of GTP from FtsZ-bound GDP by NDK. For this purpose, the ^32^P-GTP-FtsZ formed in the shortest practically monitorable time of 30 sec was immediately UV-crosslinked and quantitated. The formation of ^32^P-GTP-FtsZ was effected in two different ways: (i). 8.6 µM GDP-precharged MtFtsZ was incubated with α^32^P-GTP, for exchange with the GDP on the GDP-precharged MtFtsZ; (ii). 8.6 µM GDP-precharged MtFtsZ was incubated with γ^32^P-ATP and 0.1 µM NDK, wherein the γ^32^P-GTP-FtsZ would be formed by the NDK-mediated direct transfer of γ^32^P from γ^32^P-ATP to the GDP on the GDP-precharged MtFtsZ or by the phosphorylation of the GDP dissociated from the GDP-precharged MtFtsZ, to bind back FtsZ or exchange with the yet undissociated GDP. The assumption here is that the two modes of formation of ^32^P-GTP-FtsZ can be distinguished if one of the modes is slower than the other, with atleast one of them taking more than 30 sec to generate γ^32^P-GTP-FtsZ.

The formation of γ^32^P-GTP from γ^32^P-ATP and GDP by MsNDK, and its binding to FtsZ, along with the formation of the autophosphorylated ^32^P-NDK reaction intermediate, were verified on the SDS-PAGE for the presence of UV-crosslinked γ^32^P-GTP-MtFtsZ formed from the 30 sec reaction (S16A Fig), and on the corresponding coomassie blue stained profile for the proteins (S16B Fig). Comparable extents of the presence of ^32^P-GTP were observed on γ^32^P-GTP-MtFtsZ within the 30 sec of the addition of: (i). α^32^P-GTP to GDP-precharged MtFtsZ and (ii). γ^32^P-ATP to GDP-precharged MtFtsZ in the presence of MtNDK (S16C Fig, bars 1 and 2 in the right panel and lanes 1 and 2 in the left and middle panels). When the sample (ii), without UV-crosslinking, was boiled and loaded onto TLC plate, the formation of γ^32^P-GTP could be noted within the 30 sec of incubation of GDP-precharged MtFtsZ and γ^32^P-ATP with MtNDK or MsNDK (S16D Fig, lanes 1 and 3 in the TLC, respectively). The MsNDK-H117Q and MtNDK-H117Q could synthesise only negligible levels of γ^32^P-GTP from γ^32^P-ATP and GDP (S16D Fig, lanes 2 and 4 in the TLC, respectively). Consequentially, the extent of γ^32^P-GTP binding to MtFtsZ was also negligible (S16E Fig, bars 2 and 4 in the bar graph, respectively).

Substituting α-^32^P-ATP for γ^32^P-ATP did not show any ^32^P-GTP binding to FtsZ (S16E Fig, position 6 in the bar graph). Concurrently, the formation of α^32^P-ADP, due to the transfer of unlabeled γ-phosphate of α^32^P-ATP to GDP, could be noted (S16D Fig, lane 6 in TLC). These observations further confirmed that the GDP phosphorylation by NDK was mediated through the transfer of γ^32^P of γ^32^P-ATP to GDP to form γ^32^P-GTP. When GDP-depleted FtsZ was incubated with γ^32^P-ATP and MtNDK, in the absence of any free GDP, γ^32^P-GTP formation was not observed (S16D Fig, lane 5 in the TLC) and consequentially no γ^32^P-GTP was found on the MtFtsZ either (S16E Fig, position 5 in the bar graph).

NDK phosphorylates the FtsZ-bound GDP in the absence of free GDP

TLC showed the γ^32^P-GTP formed within 30 sec when GDP-precharged MtFtsZ was incubated with MtNDK and γ^32^P-ATP (in the absence of 1 mM exogenous GDP) (S18A Fig, lane 1, in the left panel). The γ^32^P-GTP formation could be observed also when GDP-predepleted MtFtsZ was incubated with exogenously supplied 1 mM GDP, MtNDK, and γ^32^P-ATP (S18A Fig, lane 4, in the left panel). However, the extent of γ^32^P-GTP formed in the case of GDP-predepleted MtFtsZ sample (in the presence of 1 mM exogenous GDP) was only about 60% of the γ^32^P-GTP formed in the case of GDP-precharged MtFtsZ sample (in the absence of 1 mM exogenous GDP) (S18A Fig, compare bar 4 with bar 1, in the bar graph). Interestingly, the amount of the γ^32^P-GTP formed when the GDP-precharged MtFtsZ was incubated with MtNDK and γ^32^P-ATP, in the presence of 1 mM exogenous GDP, was also only about 60% of the γ^32^P-GTP formed in the case of GDP-precharged MtFtsZ sample in the absence of 1 mM exogenous GDP (S18A Fig, compare lane 2 with lane 4 in the TLC, and bar 3 with 4 in the bar graph, respectively). The negative control, wherein the GDP-predepleted MtFtsZ was incubated with MtNDK and γ^32^P-ATP (in the absence of 1 mM GDP) did not show any γ^32^P-GTP formation (S18A Fig, lane 3 in the TLC, and bar 2 in the bar graph). These experiments showed that NDK phosphorylates free GDP preferentially over GDP bound to FtsZ, if free GDP is present along with GDP-precharged FtsZ. In corollary, NDK does phosphorylate the GDP bound to FtsZ, in the absence of free GDP in solution, more readily than it does on free GDP.

Corroborating the TLC profile on the γ^32^P-GTP formation, the SDS-PAGE profile of γ^32^P-GTP-FtsZ formation showed that γ^32^P-GTP was present on the GDP-precharged MtFtsZ, in the absence of exogenous GDP, but not in the presence of exogenous GDP (S18B Fig, lanes 1 and 2 in the left panel, and bar positions 1 and 2 in the bar graph). It showed that the γ^32^P-GTP formed from the exogenous GDP might not yet have got exchanged with the GDP bound to the GDP-precharged MtFtsZ in 30 sec, even though the GTP formed amounted to about 60% of the GTP formed in the case of GDP-precharged MtFtsZ in the absence of GDP (S18A Fig, compare bar 3 with bar 1). Similarly, the γ^32^P-GTP was not found on the GDP-predepleted MtFtsZ (in the presence of 1 mM GDP) in 30 sec (S18B Fig, lane 4 in the left panel, and bar position 4 in the bar graph). Again, it implied that the γ^32^P-GTP formed from the exogenous GDP might not yet have bound to the GDP-predepleted MtFtsZ in 30 sec, even though the γ^32^P-GTP formed amounted to about 60% of the GTP formed in the case of GDP-precharged MtFtsZ in the absence of GDP. The autophosphorylated intermediate of NDK could be seen in the absence of GDP (S18B Fig, left panel, lane 1) but not in the presence of exogenous GDP (S18B Fig, left panel, lanes 2 and 4). It showed that the phosphate group was transferred through the formation of the high energy NDK-phosphate intermediate, as it occurs in the NDK reaction. The negative control, where GDP-predepleted MtFtsZ was incubated in the presence of MtNDK and γ^32^P-ATP, did not show γ^32^P-GTP on the MtFtsZ (S18B Fig, lane 3 in the left panel, and bar position 3 in the right panel in S18B Fig).
