# Supplementary figures and images for: NDK Interacts with FtsZ and Converts GDP to GTP to Trigger FtsZ Polymerisation - A Novel Role for NDK
Source: PLoS One. 2015 Dec 2;10(12):e0143677. doi: 10.1371/journal.pone.0143677 (PMC4668074; doi:10.1371/journal.pone.0143677)

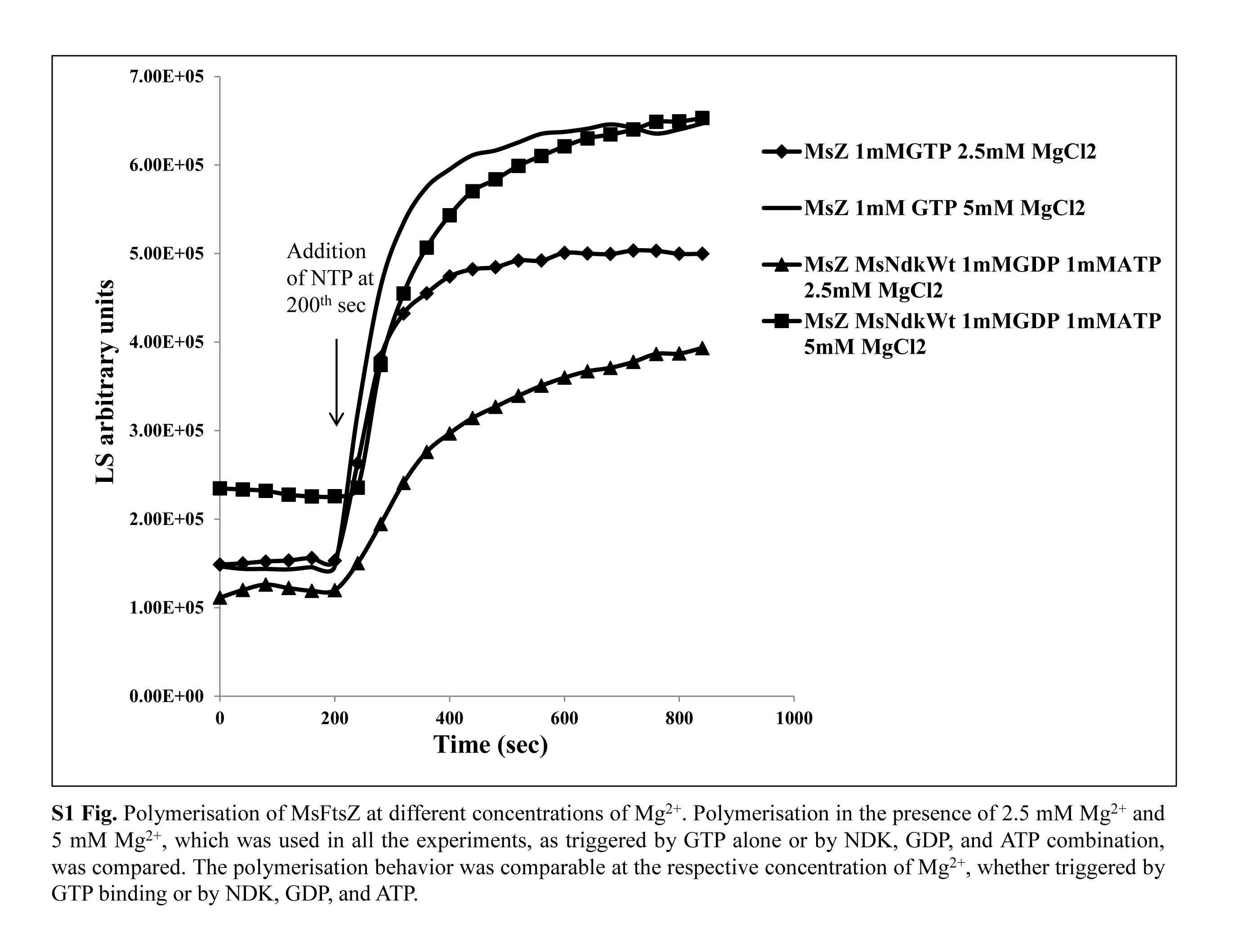

Supplement: S1 Fig — (TIF) [file pone.0143677.s002.tif]

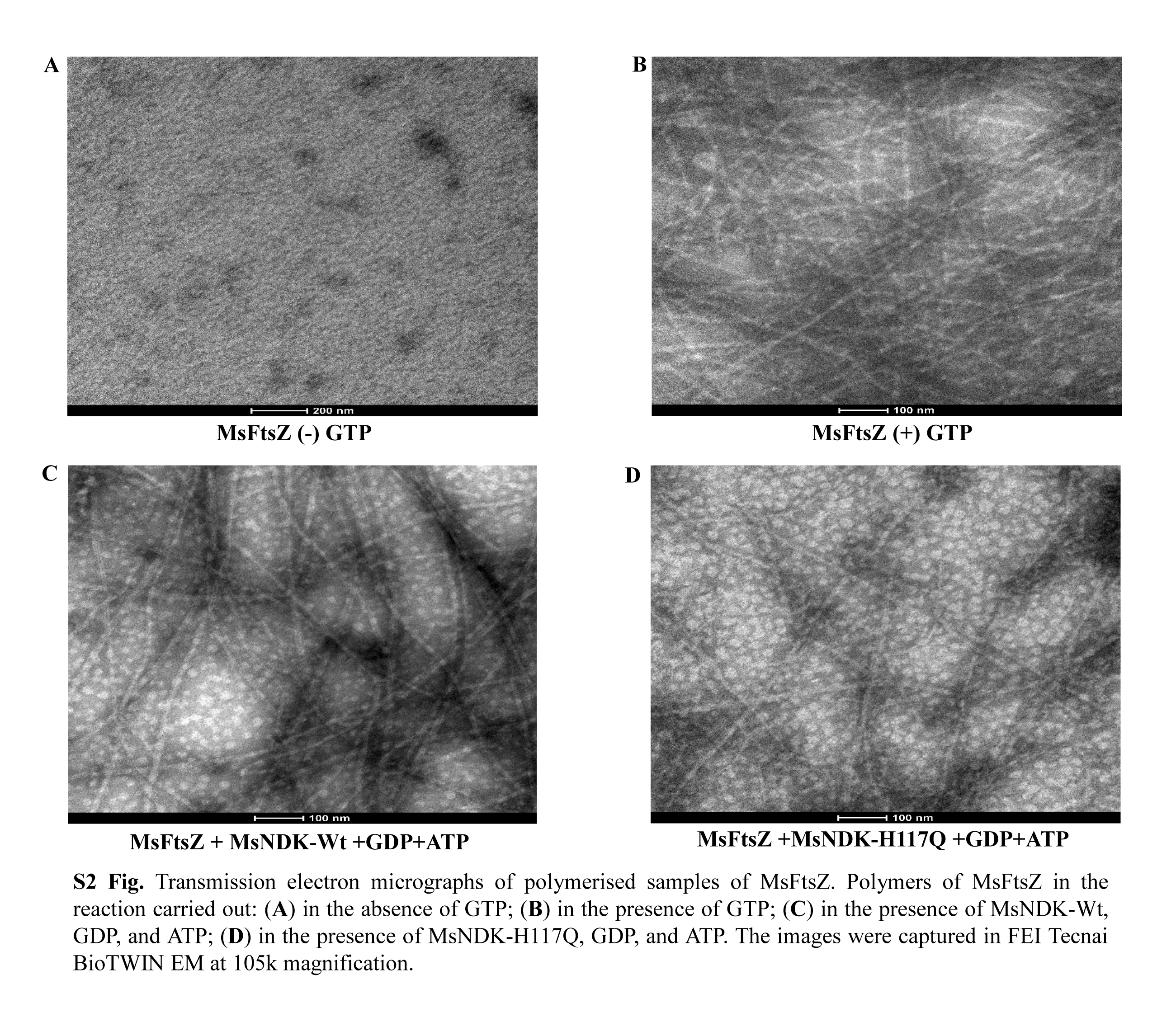

Supplement: S2 Fig — (TIF) [file pone.0143677.s003.tif]

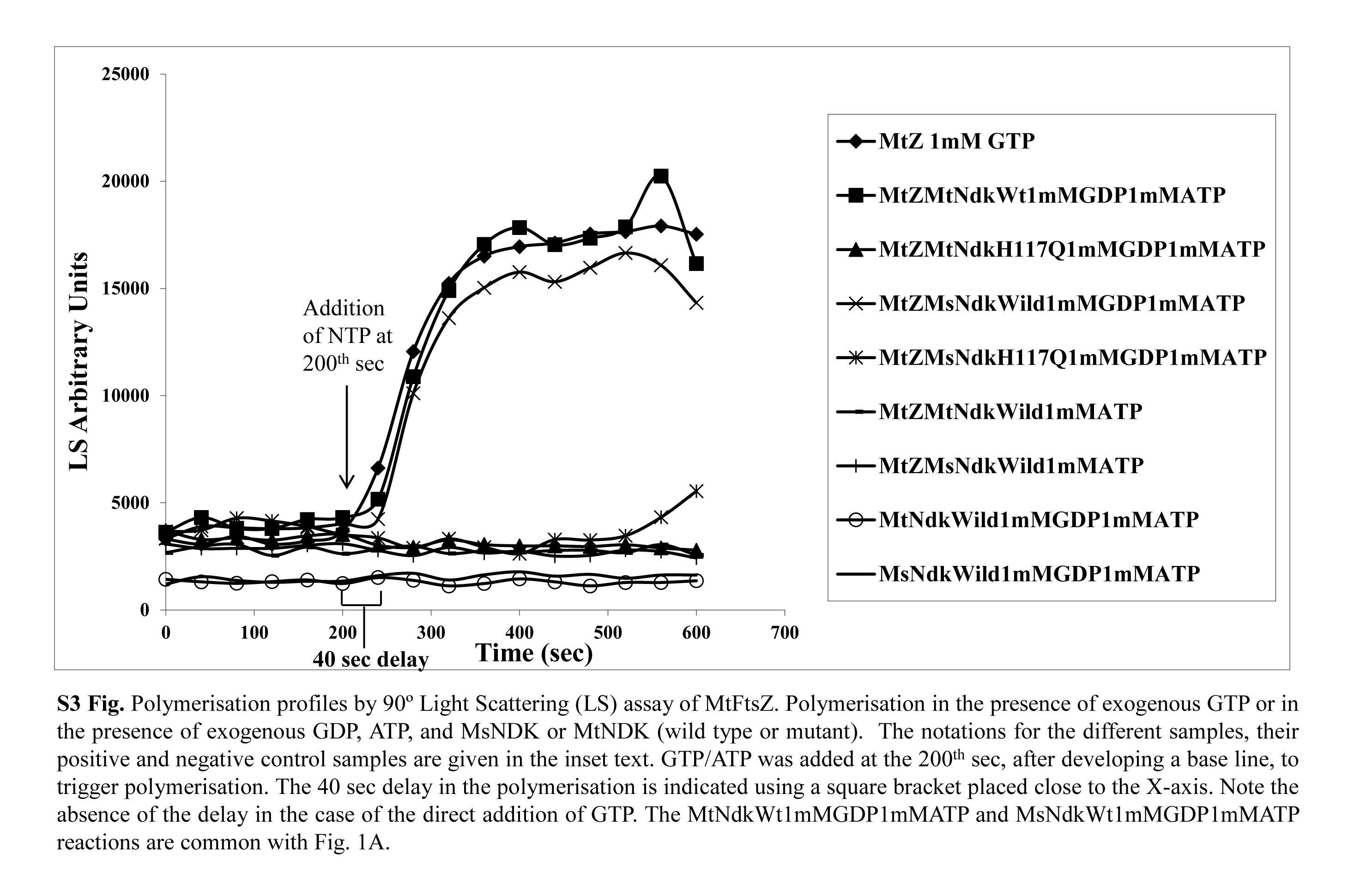

Supplement: S3 Fig — (TIF) [file pone.0143677.s004.tif]

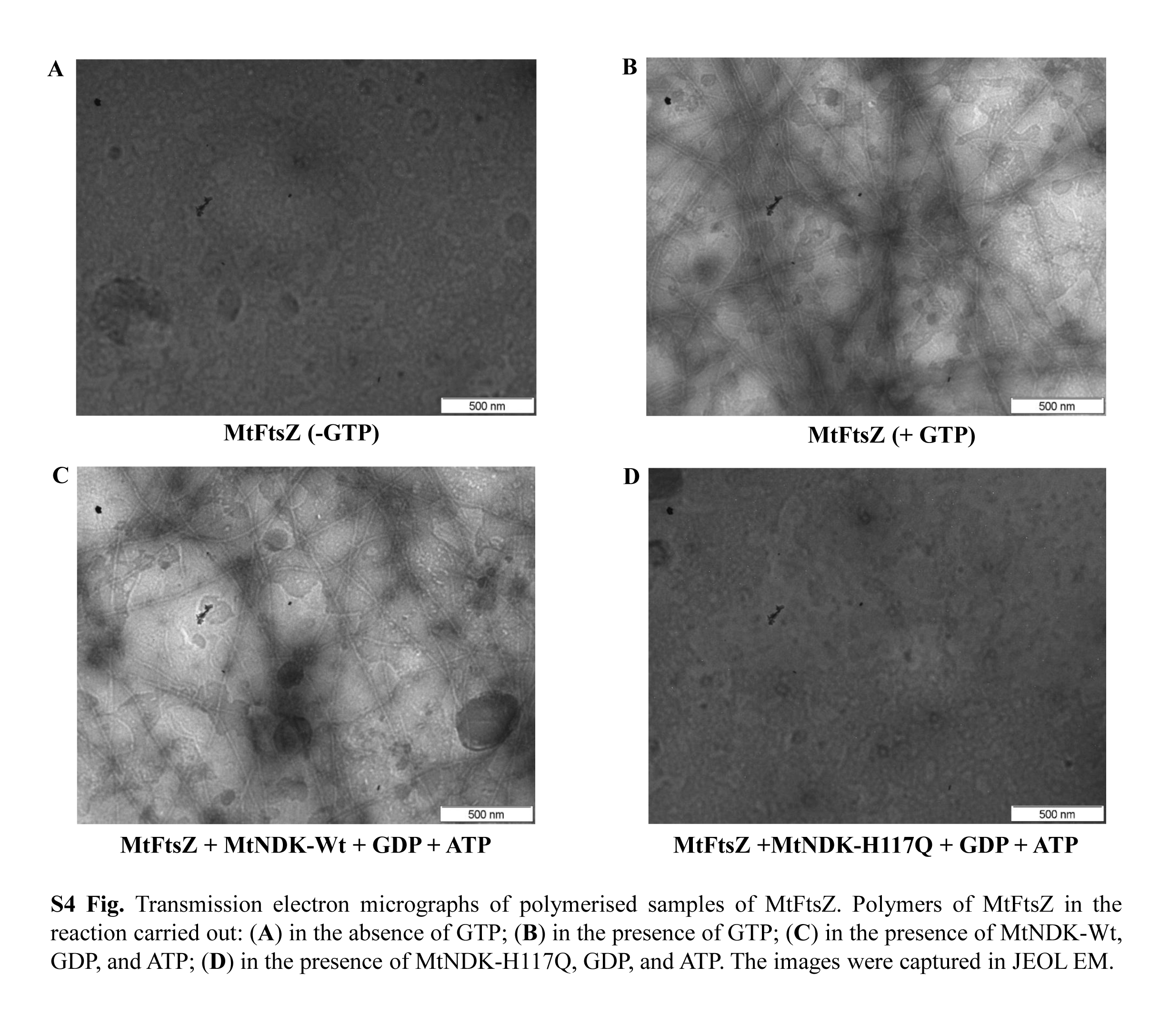

Supplement: S4 Fig — (TIF) [file pone.0143677.s005.tif]

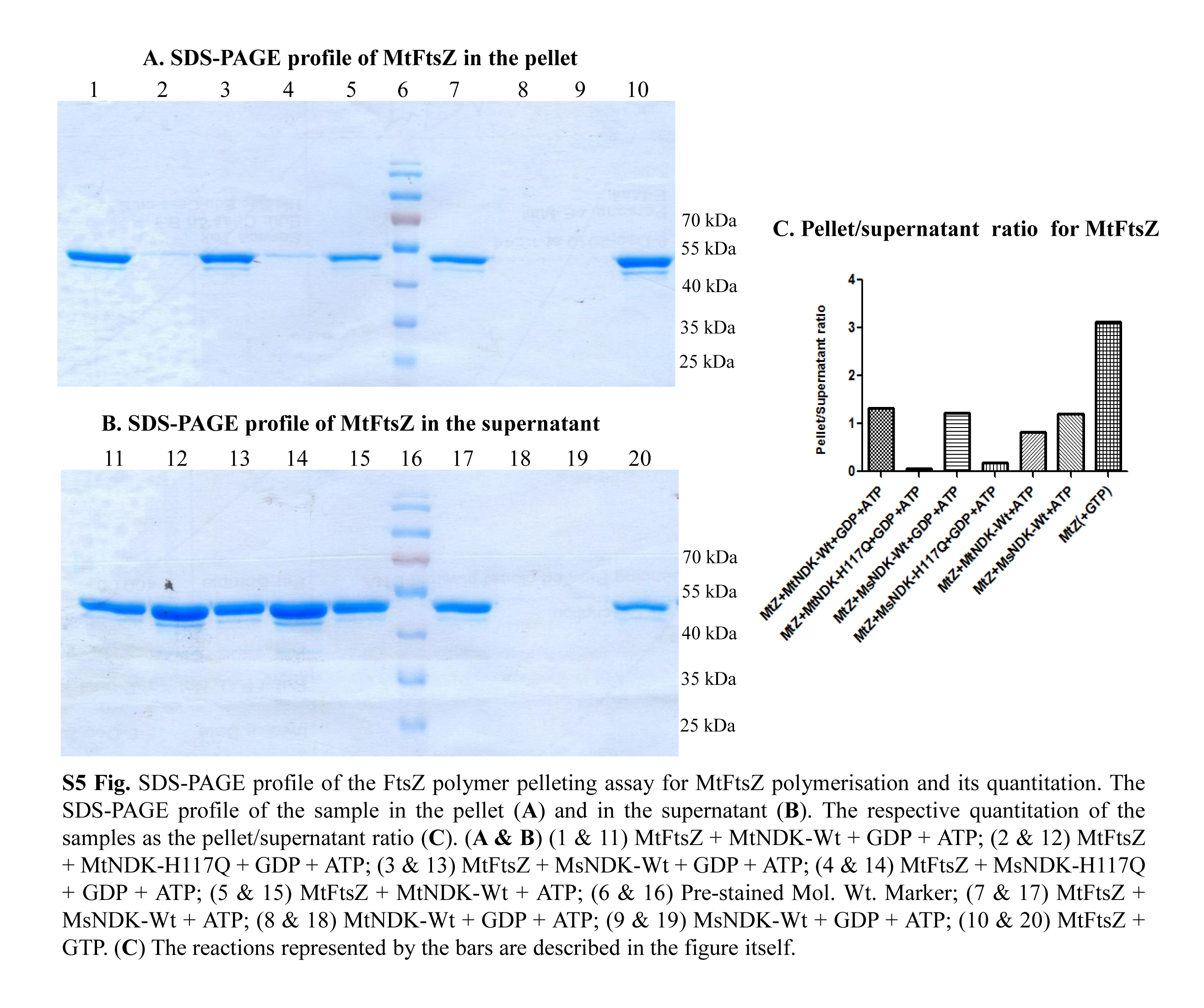

Supplement: S5 Fig — (TIF) [file pone.0143677.s006.tif]

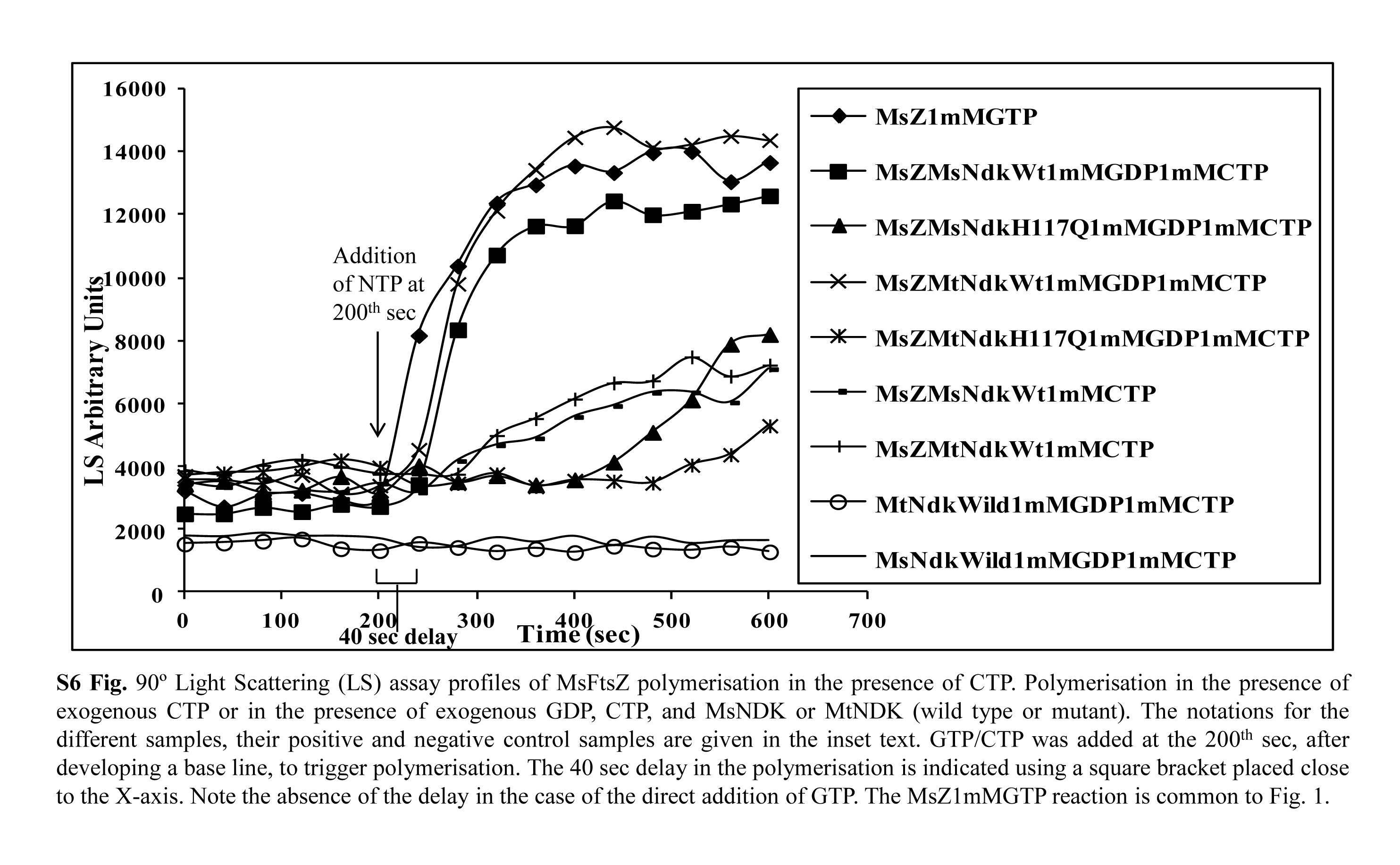

Supplement: S6 Fig — (TIF) [file pone.0143677.s007.tif]

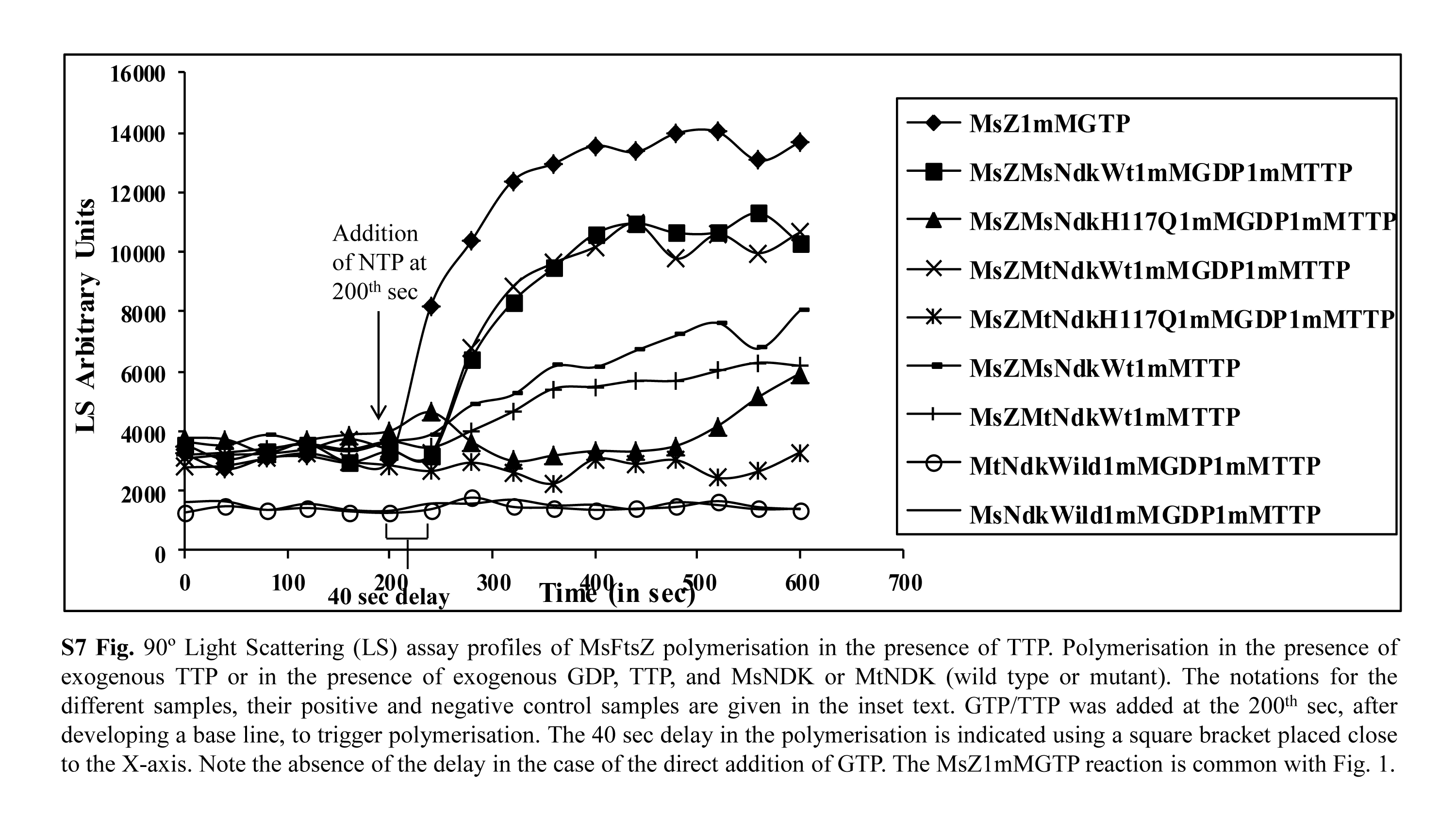

Supplement: S7 Fig — (TIF) [file pone.0143677.s008.tif]

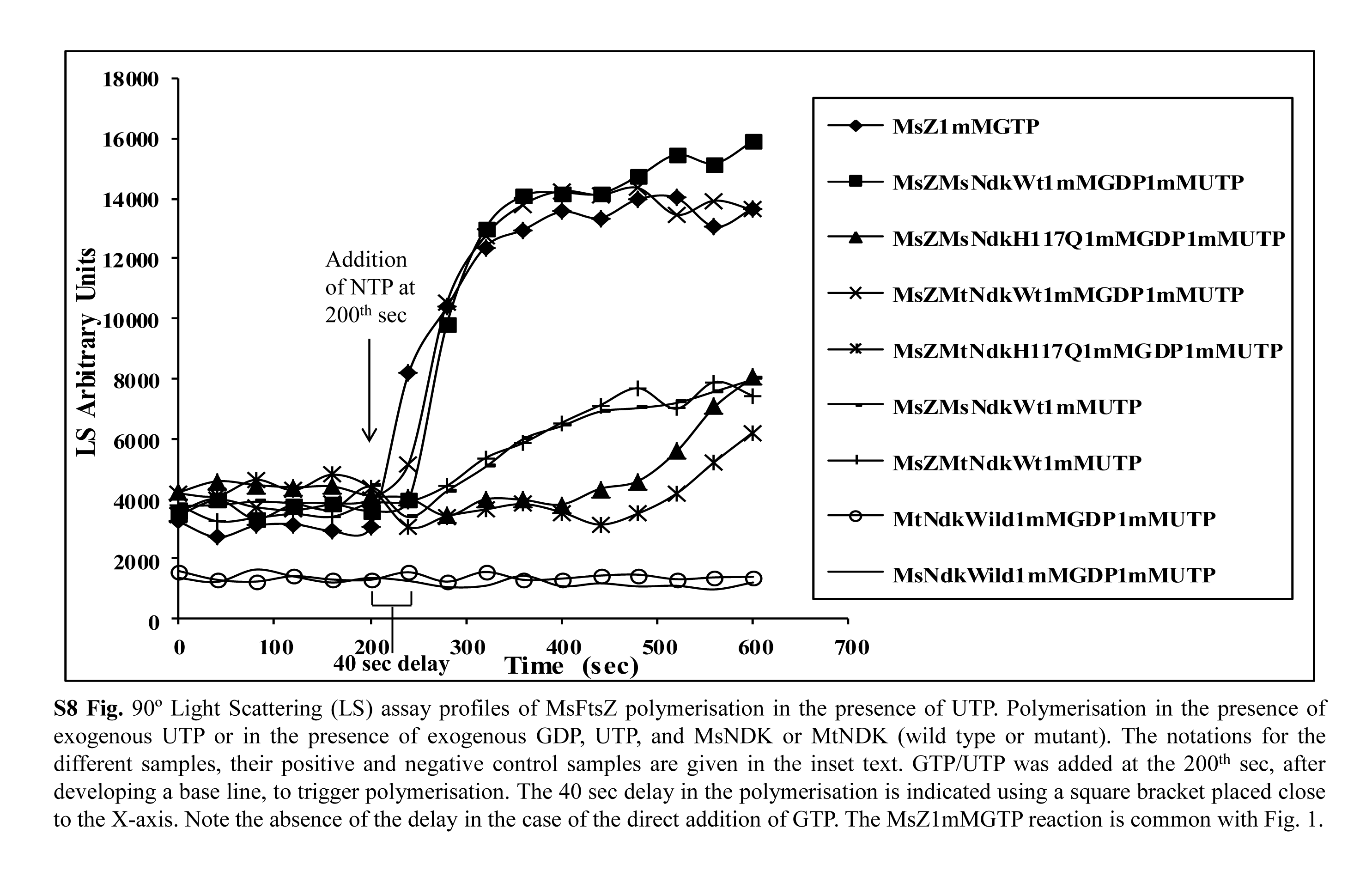

Supplement: S8 Fig — (TIF) [file pone.0143677.s009.tif]

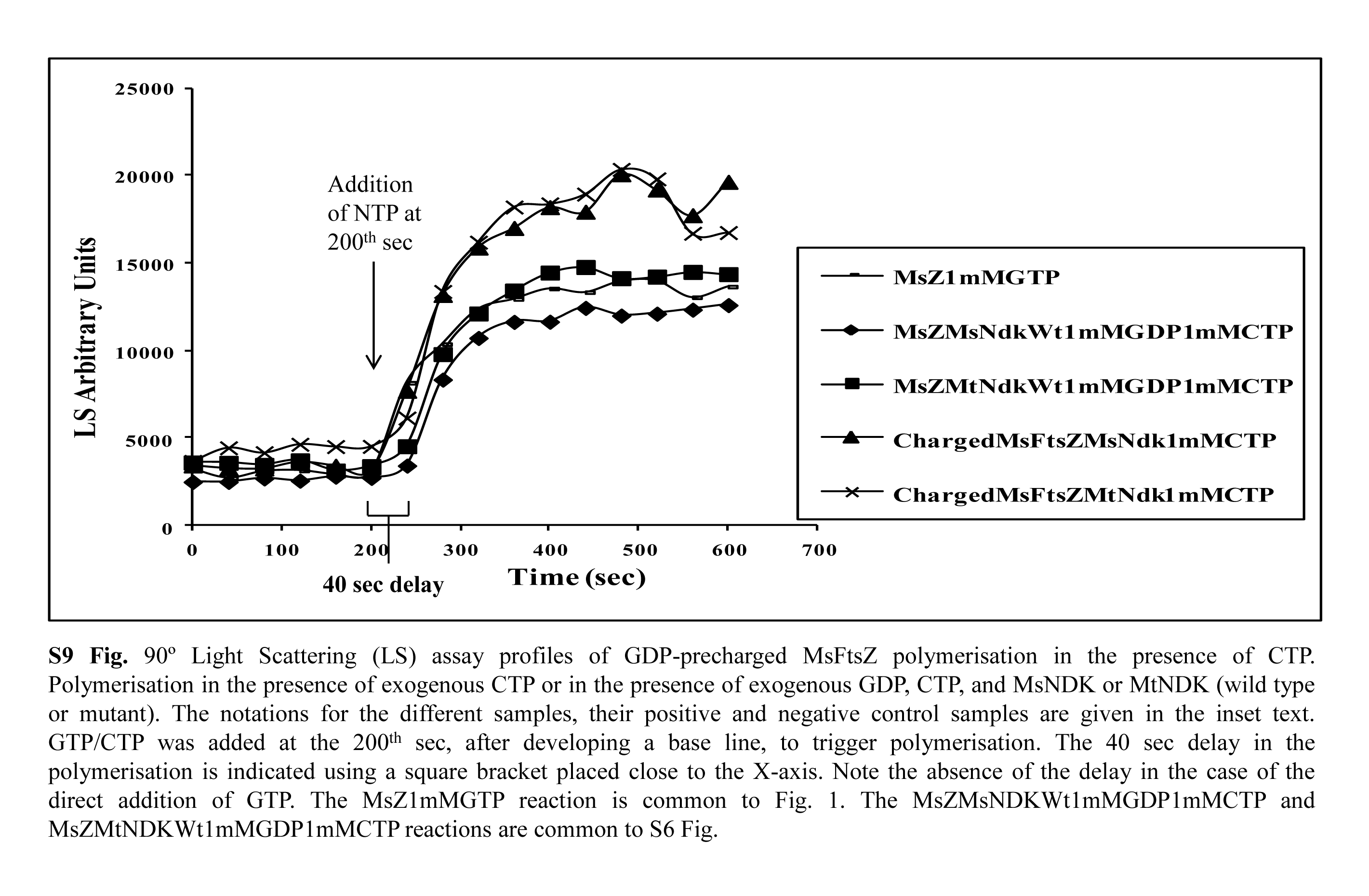

Supplement: S9 Fig — (TIF) [file pone.0143677.s010.tif]

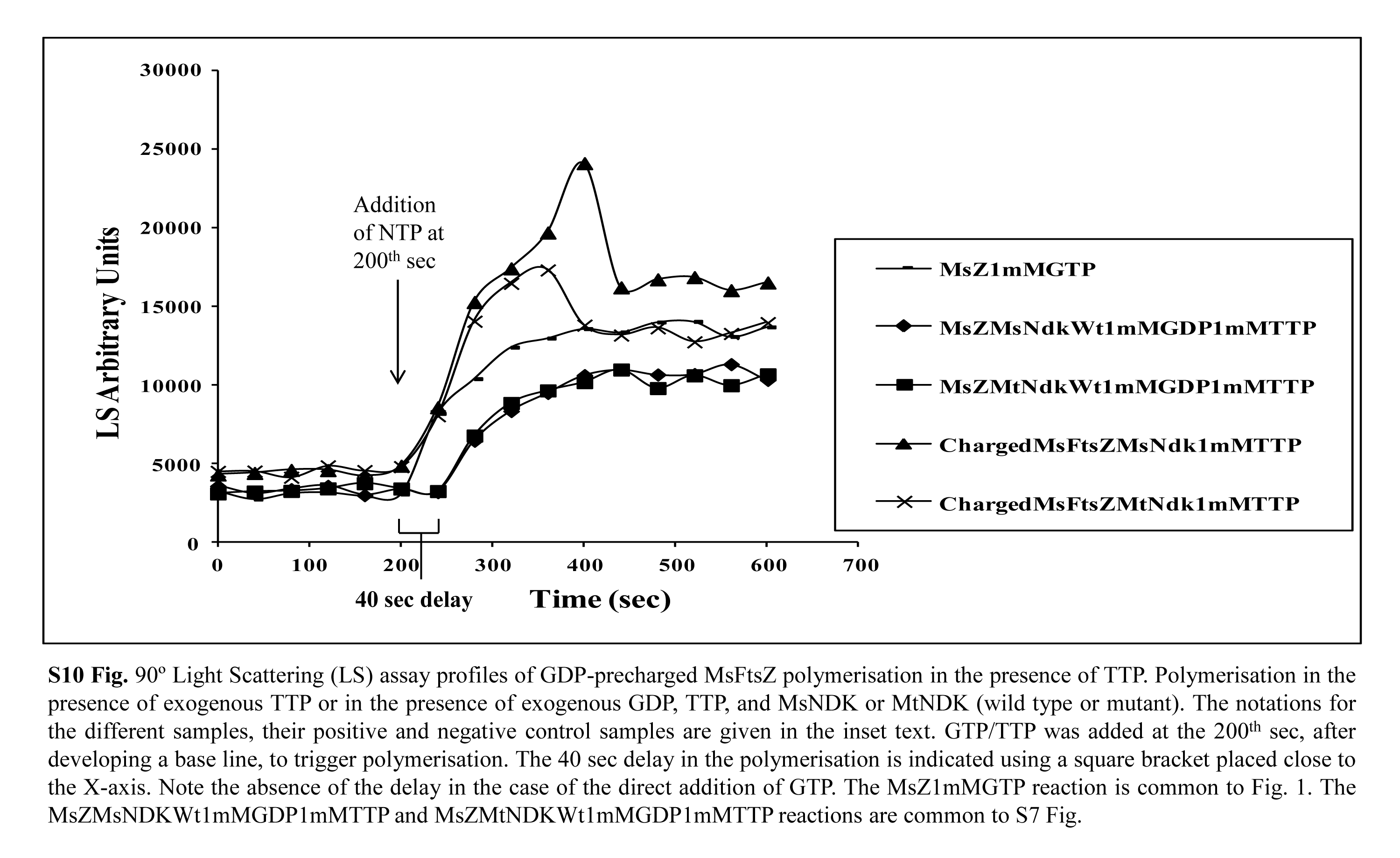

Supplement: S10 Fig — (TIF) [file pone.0143677.s011.tif]

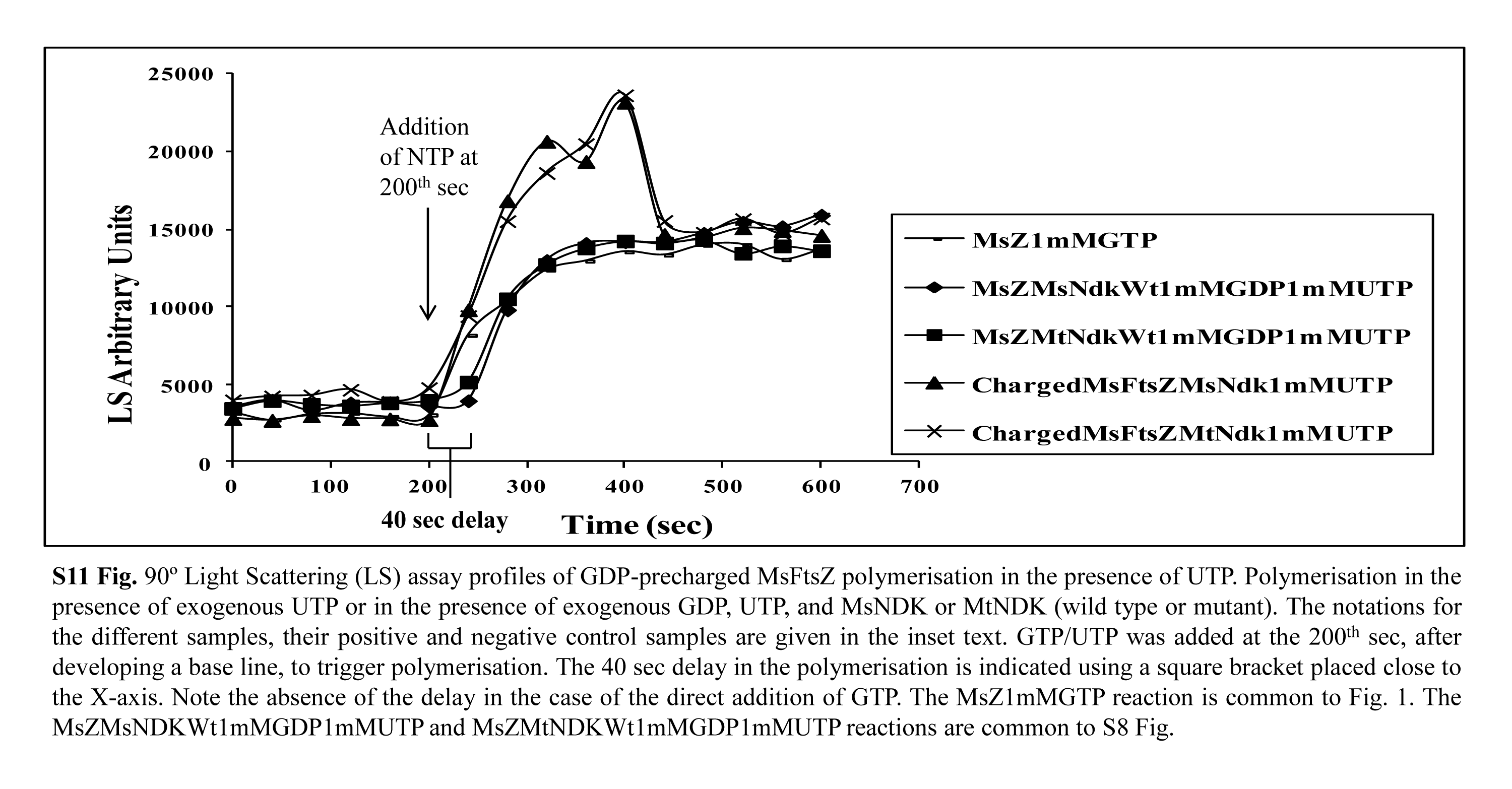

Supplement: S11 Fig — (TIF) [file pone.0143677.s012.tif]

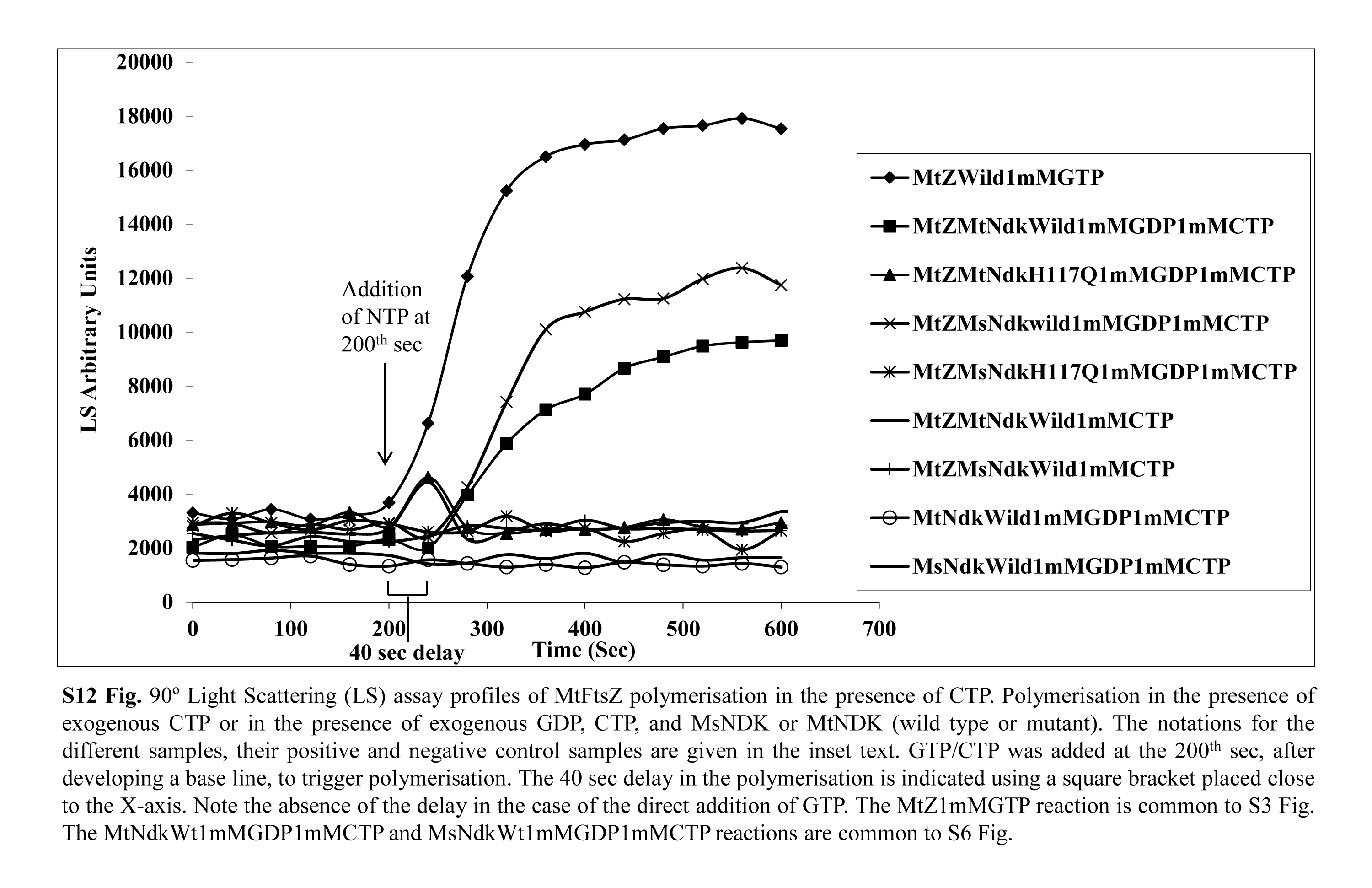

Supplement: S12 Fig — (TIF) [file pone.0143677.s013.tif]

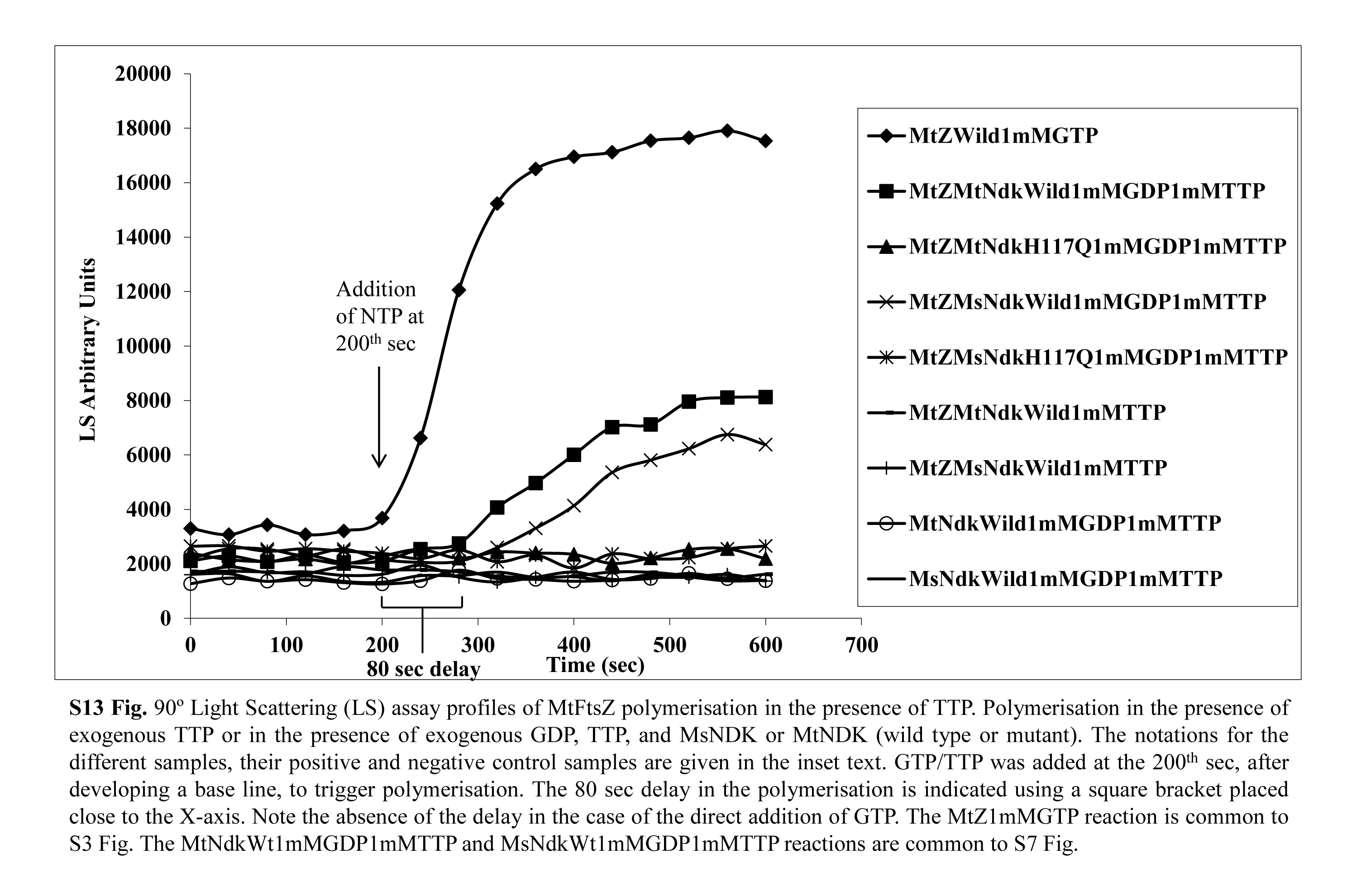

Supplement: S13 Fig — (TIF) [file pone.0143677.s014.tif]

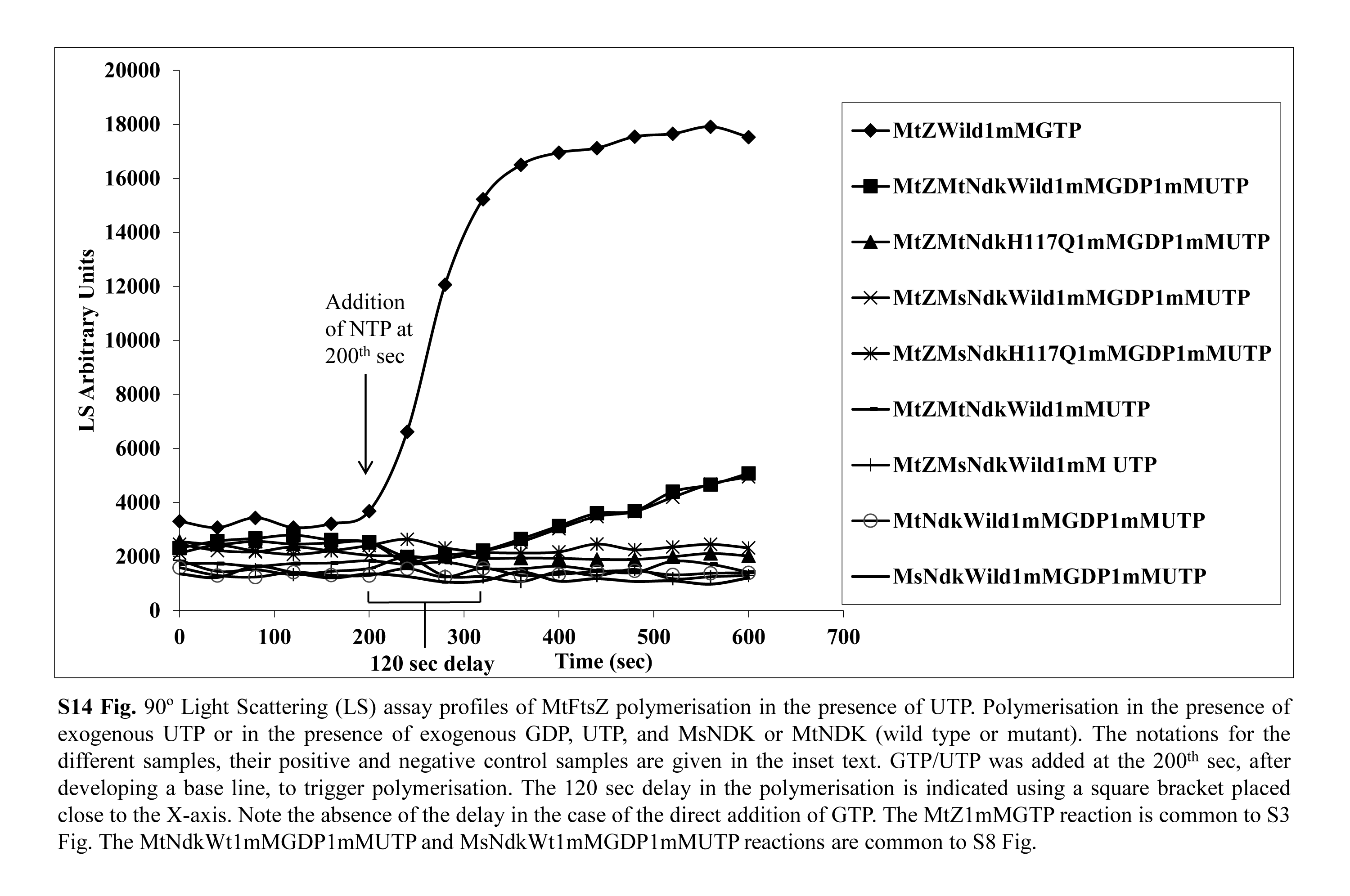

Supplement: S14 Fig — (TIF) [file pone.0143677.s015.tif]

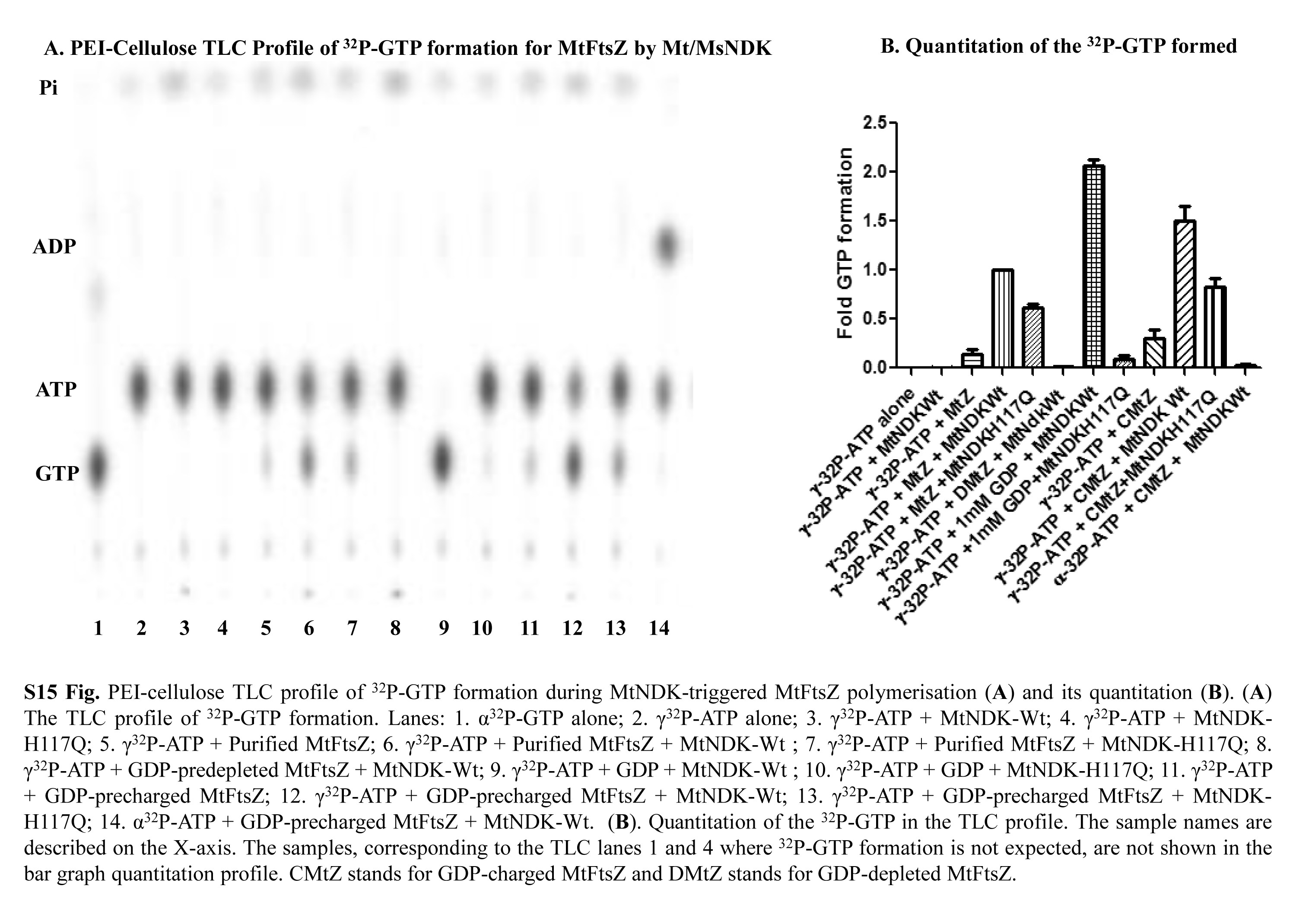

Supplement: S15 Fig — (TIF) [file pone.0143677.s016.tif]

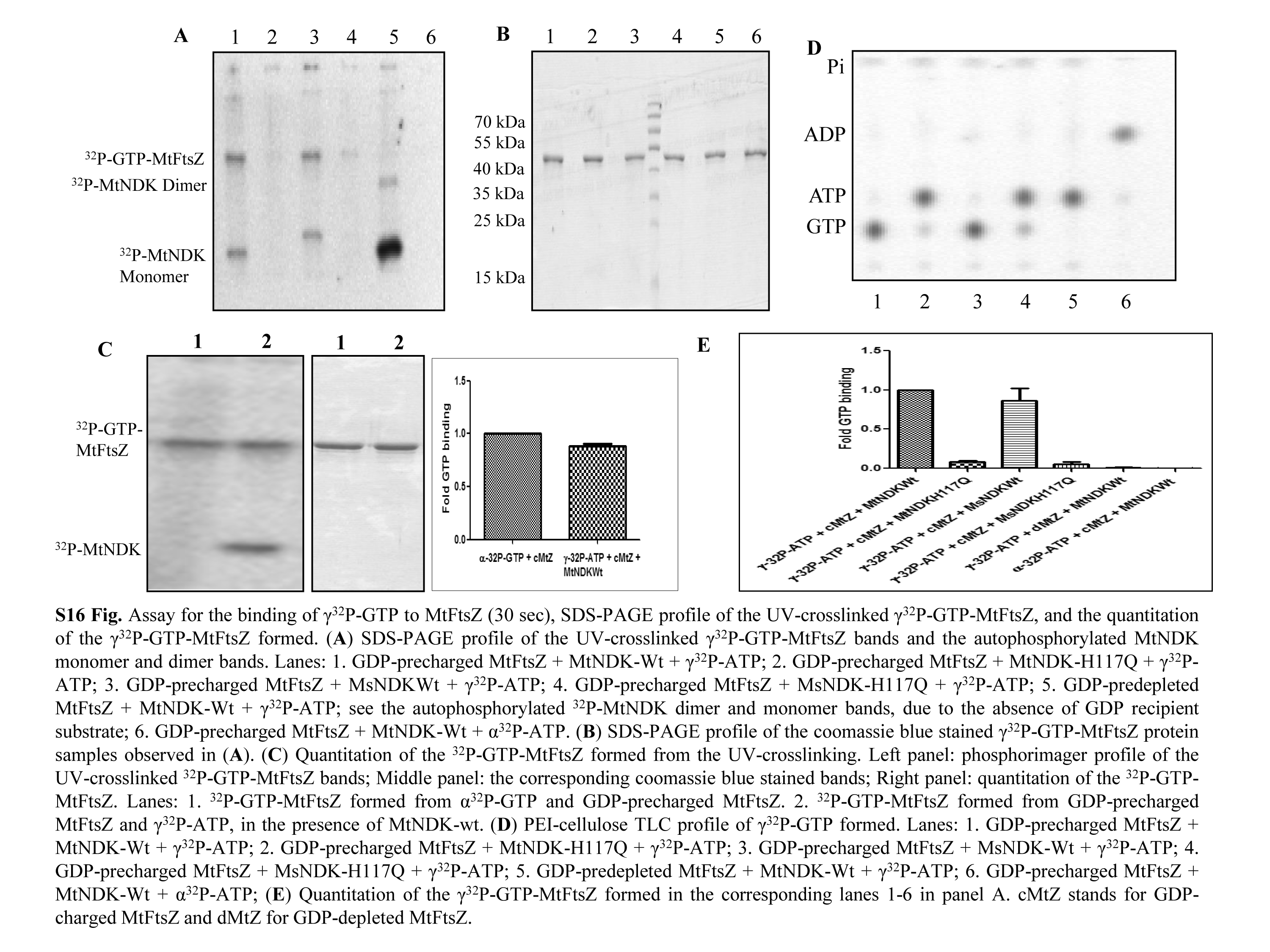

Supplement: S16 Fig — (TIF) [file pone.0143677.s017.tif]

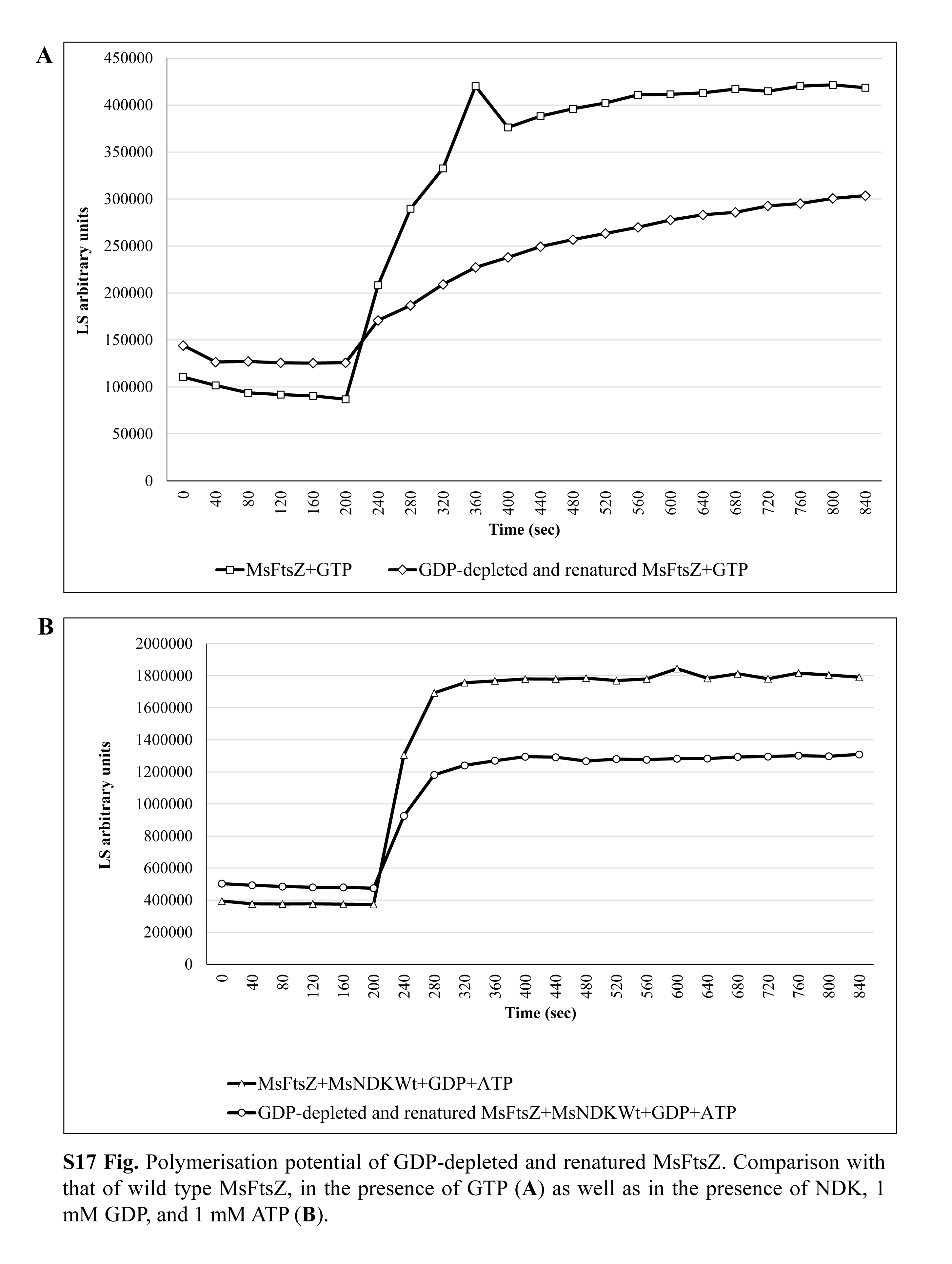

Supplement: S17 Fig — (TIF) [file pone.0143677.s018.tif]

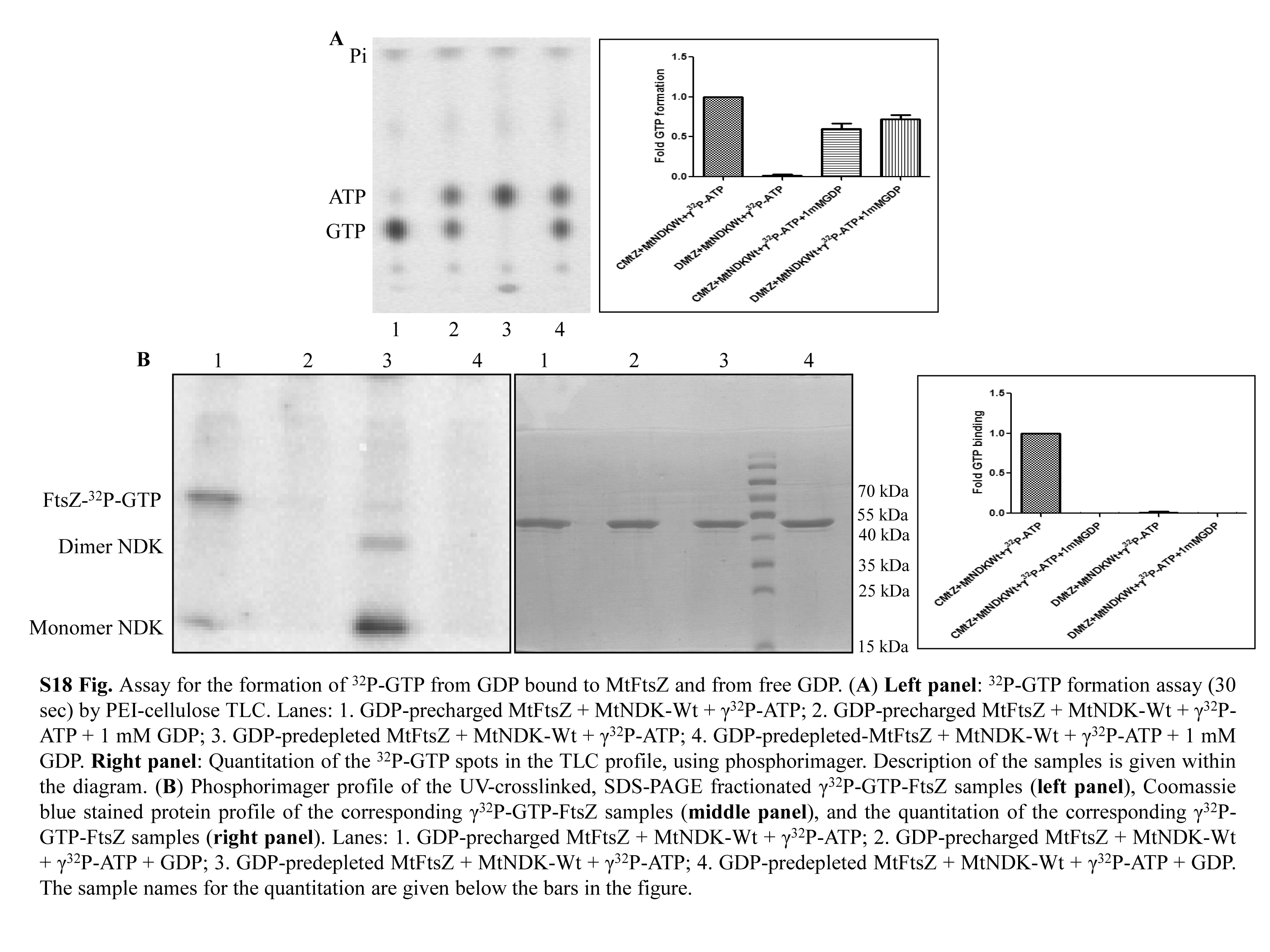

Supplement: S18 Fig — (TIF) [file pone.0143677.s019.tif]
