# Supplementary material for: NDK Interacts with FtsZ and Converts GDP to GTP to Trigger FtsZ Polymerisation - A Novel Role for NDK
Source: PLoS One. 2015 Dec 2;10(12):e0143677. doi: 10.1371/journal.pone.0143677 (PMC4668074; doi:10.1371/journal.pone.0143677)
Supplement: S1 Table — (DOCX) [file pone.0143677.s020.docx]

**S1 Table. List of bacterial strains**

Name Relevant Genotype Reference or Source

*E. coli* C41 BL21 (DE3) derivative, a strain J. E. Walker [1]

used for expression of toxic proteins

*E. coli* JM109 [*Δ (lac-pro) endA1 recA1 hsdR17* [2]

*thi relA gyrA96 supE44/F' lacI^q^*

*traD36 proAB+ lacZΔ M15*]

*E. coli* M15 *E. coli* K12 derivative, *thi^-^ lac^-^ ara^+^*  Qiagen

*lon^-^ recA^+^ uvr^+^*

*M. smegmatis* mc^2^155 William R. Jacobs [3]

*M. tuberculosis* H_37_R_a_ JALMA Institute of

Leprosy and Other

Mycobacterial

Diseases, Agra, India

**References**

1. Miroux B, Walker JE. Over-production of proteins in Escherichia coli: mutant hosts that allow synthesis of some membrane proteins and globular proteins at high levels. Journal of Molecular Biology. 1996;260(3):289-98. doi: 10.1006/jmbi.1996.0399. PubMed PMID: 8757792.

2. Yanisch-Perron C, Vieira J, Messing J. Improved M13 phage cloning vectors and host strains: nucleotide sequences of the M13mp18 and pUC19 vectors. Gene. 1985;33(1):103-19. PubMed PMID: 2985470.

3. Snapper SB, Melton RE, Mustafa S, Kieser T, Jacobs WR, Jr. Isolation and characterization of efficient plasmid transformation mutants of *Mycobacterium smegmatis*. Molecular Microbiology. 1990;4(11):1911-9. PubMed PMID: 2082148.
