# Supplementary material for: NDK Interacts with FtsZ and Converts GDP to GTP to Trigger FtsZ Polymerisation - A Novel Role for NDK
Source: PLoS One. 2015 Dec 2;10(12):e0143677. doi: 10.1371/journal.pone.0143677 (PMC4668074; doi:10.1371/journal.pone.0143677)
Supplement: S2 Table — (DOCX) [file pone.0143677.s021.docx]

**S2 Table.** **Oligonucleotide primers**

MtNDK1 5’ gggggatccgtgaccgaacggactctggtactgatcaag 3’

MtNDK2 5’ ccggaattcggcgccgggaaaccagagcg 3’

T3 5' aattaaccctcactaaaggg 3'

H117Q1 5’ gttcaacctggtgca**g**ggatccgattcggccgaatccgcg 3’

H117Q2 5’ cgcggattcggccgaatcggatcc**c**tgcaccaggttgaac 3’

T7 5' gtaatacgactcactatagggc 3'

Mt1 5’ cgggatcccatatgacccccccgcacaactacctggccgtcatcaag 3’

MtftsZ-C2 5' gcggcgcatgaagggcggcacgtcgacatc 3'

MsZf1 5’ gcgggatccgatatcatgacccccccgcac 3’

MsZr1 5’ gcgtctagagaattcgtgccgcatgaagggcggc 3’

Restriction enzyme sites are underlined and mutations introduced are given in bold letters.
