# Supplementary material for: NDK Interacts with FtsZ and Converts GDP to GTP to Trigger FtsZ Polymerisation - A Novel Role for NDK
Source: PLoS One. 2015 Dec 2;10(12):e0143677. doi: 10.1371/journal.pone.0143677 (PMC4668074; doi:10.1371/journal.pone.0143677)
Supplement: S3 Table — (DOCX) [file pone.0143677.s022.docx]

**S3 Table. List of the plasmid vectors**

**_______________________________________________________________________Name** **Relevant Feature** **Reference or Source**

pCR-SCRIPT (SK) Cloning vector Stratagene

pCR-SCRIPT- pCR-SCRIPT carrying This Study

-MtNDK MtNDK ORF

pQE30 Cloning/expression vector with Qiagen

T5 promoter & N-terminal 6xHis tag

pQE30-MtNDK pQE30 carrying 6xHis-MtNDK This study

pENTR3C Cloning vector Invitrogen

pENTR3C-MtNDK pENTR3C carrying MtNDK This study

pENTR3C-MtFtsZ pENTR3C carrying MtFtsZ This study

pDEST15 Cloning vector with N-terminal GST tag Invitrogen

pDEST15-MtNDK pDEST15 carrying GST-MtNDK This study

pCR-SCRIPT- pCR-SCRIPT carrying This Study

-MtNDK-H117Q MtNDK-H117Q ORF

pQE30-MtNDK-H117Q pQE30 carrying 6xHis-MtNDK-H117Q This study

pDEST17 Cloning vector with N-terminus 6xHis tag Invitrogen

pDEST17-MtFtsZ pDEST17 carrying 6xHis-MtFtsZ This study

pBlueScript (pBS) Cloning/Expression vector Stratagene

pBS-KS-MtFtsZ pBS-KS carrying MtFtsZ ORF This study

pET15b+ Expression vector with T7 promoter Novagen

and N-terminal 6xHis tag

pET15b-MsFtsZ pET15b+ carrying MsFtsZ ORF This study
