# Supplementary material for: NDK Interacts with FtsZ and Converts GDP to GTP to Trigger FtsZ Polymerisation - A Novel Role for NDK
Source: PLoS One. 2015 Dec 2;10(12):e0143677. doi: 10.1371/journal.pone.0143677 (PMC4668074; doi:10.1371/journal.pone.0143677)
Supplement: S1 Text — (DOCX) [file pone.0143677.s023.docx]

**S1 Text (Supplementary Text)**

**NDK Interacts with FtsZ and Converts GDP to GTP to Trigger FtsZ polymerisation –**

**A Novel Role for NDK**

Saurabh Mishra^1^, Kishor Jakkala^1^, Ramanujam Srinivasan^1,#a^, Muthu Arumugam^1,#b^, Raghavendra Ranjeri^1^, Prabuddha Gupta^1,#c^, Haryadi Rajeswari^1^, and Parthasarathi Ajitkumar^1,^*

**Materials and Methods**

pQE30-MtNDK: MtNDK ORF was PCR amplified from *M. tuberculosis* H_37_Ra genomic DNA, using MtNDK1 and MtNDK2 primers (S2 Table), digested with BamHI and EcoRI and ligated to pCR-SCRIPT (SK) (Stratagene), digested with the same enzymes. The resulting construct (pCR-SCRIPT-MtNDK) was digested with BamHI and HindIII. The released fragment was the ligated to pQE30, digested with BamHI and HindIII, to yield pQE30-MtNDK.

GST-MtNDK and pQE30-MtNDK-H117Q: pCR-SCRIPT-MtNDK was digested with BamHI and EcoRI and ligated to pENTR3C digested with the same enzymes to obtain pENTR3C-MtNDK. The MtNDK ORF was then subcloned from pENTR3C-MtNDK into pDEST15 using LR Clonase, to yield pDEST15-MtNDK. pQE30-MtNDK-H117Q was constructed by overlapping PCR on pCR-SCRIPT-MtNDK using T3 and H117Q2 primers (amplifies 5’ end) (S2 Table) and then with H117Q1 and T7 primers (amplifies 3’ end) (S2 Table). The PCR products were further amplified with T3 and T7 primers. The resulting PCR product was digested with SmaI and HindIII and ligated to pCR-SCRIPT (Stratagene) to obtain pCR-SCRIPT-MtNDK-H117Q, which was sequence verified, then digested with SmaI and HindIII, and the fragment was ligated at the same sites in pQE30 to obtain pQE30-MtNDK-H117Q.

pDEST17-MtFtsZ and pET15b-MtftsZ: MtFtsZ ORF was PCR amplified from *M. tuberculosis* H_37_R_a_ genomic DNA, using Mt1 and MtftsZ-C2 primers (S2 Table). The resulting product was digested with BamHI and ligated to BamHI and EcoRV digested pENTR3C, to obtain pENTR3C-MtFtsZ, and sequence verified. The MtFtsZ ORF was then subcloned from pENTR3C-MtFtsZ into pDEST17 using LR Clonase, to obtain pDEST17-MtFtsZ. The cloning of pET15b-MtftsZ was already described [1].

*MsftsZ*: The *ftsZ* gene of *Mycobacterium smegmatis* (MsFtsZ) was amplified from *M. smegmatis* mc^2^155 genomic DNA, using MsZf1 as the forward primer and MsZr1 as the reverse primer (S2 Table). *Pfu* DNA polymerase (Fermentas) was used for amplification, following manufacturers’ protocol. MsftsZ PCR product was cloned in pBluescript (KS) at BamH1-Xba1 sites and sequence verified on both the strands, to get pBS-KS-MsFtsZ. The pBluescript (KS) construct containing MsFtsZ ORF (pBS-KS-MsFtsZ) was digested with BamH1 and Not1, the released inserts were end-filled using Klenow DNA polymerase (NEB) according to manufacturer’s protocol, and subcloned into end-filled BamH1 site of pET15b+ to get pET15b-MsFtsZ.

*MsNDK* and *MsNDK*-H117Q: *MsNDK* gene was cloned from *M. smegmatis* mc^2^155 genomic DNA, and the mutant MsNDK-H117Q was generated, as described [2].

Purification of 6xHis-MtNDK, 6xHis-MtNDK-H117Q, and GST-MtNDK proteins

*E. coli* M15/pREP4 transformant carrying pQE30-MtNDK was grown in Luria broth containing relevant antibiotics and harvested after induction with 1 mM IPTG for 3 hrs. The cells were lysed by sonication, and 6xHis-MtNDK was purified using Ni^2+^-NTA agarose, as described [3], and dialysed against 100 mM Tris-HCl (pH 8.0), containing 50 mM KCl and 10% glycerol. Protein samples were stored in aliquots at -80°C until further use. Purification of MtNDK-H117Q was carried out under conditions identical to those of 6xHis-MtNDK. GST-MtNDK was purified essentially as described above except that *E. coli* C41 strain [4] was used for expression and glutathione-agarose beads were used for affinity purification. The protein was eluted with 50 mM Tris-HCl (pH 8.0), 100 mM KCl, 1 mM DTT, 1 mM MgCl_2_ and 10% glycerol containing 10 mM reduced glutathione. The purified protein was dialysed against 50 mM Tris–HCl (pH 8.0), 100 mM KCl, 1 mM DTT, 1 mM MgCl_2_ and 10% glycerol, and subsequently buffer exchanged against 25 mM HEPES-NaOH (pH 7.2) containing 100 mM KCl, 1 mM DTT, 1 mM MgCl_2_ and 10% glycerol, using PD10 desalting column (GE Health Sciences) when required.

Overexpression and Purification of 6xHis-MtFtsZ protein

The 6xHis-MtFtsZ was expressed from pET15b-MtFtsZ and was purified from soluble fractions, as described [5], but with minor modifications [1]. *E. coli* C41 cells [4] were transformed with the individual expression constructs and the expression-verified clones were induced with 0.5 mM IPTG (final concentration) at 0.6 OD_600_ and kept at 30°C with shaking at 170 rpm for 4 hrs. The cells were harvested and suspended in the lysis buffer (229 mM NaCl, 4.51 mM KCl, 16.7 mM Na_2_HPO_4_ and 3.34 mM KH_2_PO_4_, pH 7.8), containing 1 mM PMSF and 2 mg lysozyme and kept on ice for 30 min with occasional mixing. Cells were finally lysed by sonication using a Sonics^TM^ sonicator in five repeat sonications, each consisting of 15 sec pulse mode at 60% duty cycle at an interval of 30 sec. Soluble fraction of the lysate was recovered by centrifugation at 12000 rpm for 30 min at 4°C in a Sorvall RC-5B centrifuge, using SS-34 rotor. The soluble fraction was mixed with 1 ml bed volume of Ni^2+^-NTA agarose slurry equilibrated with lysis buffer containing 1 mM PMSF and kept for batch-binding at 4°C on a rotating platform for 3 hrs. Protein-bound agarose (1 ml bed volume, as indicated above) was collected in a BioRad Glass Econo-Column^TM^ and washed with 20 ml lysis buffer containing 1 mM PMSF and 20 mM imidazole. Finally, protein was eluted with 2 ml lysis buffer containing 1 mM PMSF and 500 mM imidazole by allowing the Ni^2+^-NTA beads to stand in the elution buffer for 30 min in the locked elution column, and subsequently eluting in 500 μl aliquotes. The eluted 6xHis-tagged protein preparation was dialysed (Pierce SnakeSkin^TM^ Pleated Dialysis Tubing, 10 kDa cutoff) in 50 mM HEPES-NaOH, pH 7.2, containing 1 mM DTT and 10% glycerol, concentrated using 2 ml volume capacity VivaScience concentrator of 10 kDa cut-off, and stored in 2-3 times usable aliquots at -80°C. This protein was used in the FtsZ polymerisation assays.

6xHis-MtFtsZ was also expressed from pDEST17-MtFtsZ and purified from soluble fraction, as described [5] with minor modifications [1]. In brief, *E. coli* C41 cells [4] harbouring pDEST17-MtFtsZ were induced with 1 mM IPTG and harvested after 3 hrs. The pellet was resuspended in 40 ml of lysis buffer [20 mM sodium phosphate buffer (pH 7.4), 500 mM NaCl, 2 mM PMSF, 4 mg/ml of pepstatin A, 4 mg/ml of leupeptin and 20 mg/ml of soyabean trypsin inhibitor] and briefly sonicated. The cell suspension was digested with 10 units of DNase I (10000 U/ml) for 30 min and then extracted by passing the suspension through French press at 15000 to 20000 lb/in^2^. The solution was clarified by centrifugation at 15000 rpm for 20 min and then applied onto Ni^2+^-NTA agarose column pre-equilibrated with 10 mM imidazole and 20 mM sodium phosphate (pH 7.4) buffer containing 500 mM NaCl. The column was washed with 20 ml of equilibration buffer containing 40 mM imidazole. Recombinant FtsZ was eluted with 250 mM imidazole in the equilibration buffer. The eluate was immediately passed over Sephadex G-25 column (PD-10 column, GE Health Sciences) equilibrated with 25 mM HEPES-NaOH (pH 7.2) containing 100 mM KCl, 0.1 mM EDTA, 1 mM dithiothreitol (DTT) and 10% glycerol. The eluate fractions containing FtsZ were pooled and concentrated. The concentrated protein was dialysed against 25 mM HEPES-NaOH (pH 7.2) buffer containing 100 mM KCl, 100 mM EDTA, 1 mM dithiothreitol (DTT) and 10% glycerol. The protein was aliquoted and stored at -80°C until further use. This protein was used in the NDK-FtsZ pulldown assays for NDK-FtsZ interaction.

Overexpression and purification of 6xHis-MsFtsZ protein

Expression of MsFtsZ soluble protein was carried out essentially, as described for MtFtsZ [5], with some modifications, similar to those mentioned for MtFtsZ [1]. In brief, *E. coli* C41 cells [4] were transformed with the expression construct (pET15b-MsFtsZ) and the expression-verified clones were induced with 0.5 mM IPTG (final concentration) at 0.6 OD_600_ and kept at 30°C with shaking at 170 rpm for 4 hrs. The cells were harvested and suspended in the lysis buffer (229 mM NaCl, 4.51 mM KCl, 16.7 mM Na_2_HPO_4_ and 3.34 mM KH_2_PO_4_, pH 7.8), containing 1 mM PMSF and 2 mg lysozyme and kept on ice for 30 min with occasional mixing. Cells were finally lysed by sonication using a Sonics^TM^ sonicator in five repeat sonications, each consisting of 15 sec pulse mode at 60% duty cycle at an interval of 30 sec. Soluble fraction of the lysate was recovered by centrifugation at 12000 rpm for 30 min at 4°C in a Sorvall RC-5B centrifuge, using SS-34 rotor. The soluble fraction was mixed with 1 ml bed volume of Ni^2+^-NTA agarose slurry equilibrated with lysis buffer containing 1 mM PMSF and kept for batch-binding at 4°C on a rotating platform for 3 hrs. Protein-bound agarose (1 ml bed volume, as indicated above) was collected in a BioRad Glass Econo-Column^TM^ and washed with 20 ml lysis buffer containing 1 mM PMSF and 20 mM imidazole. Finally, protein was eluted with 2 ml lysis buffer containing 1 mM PMSF and 500 mM imidazole by allowing the Ni^2+^-NTA beads to stand in the elution buffer for 30 min in the locked elution column, and subsequently eluting in 500 μl aliquotes. The eluted 6xHis-tagged protein preparation was dialysed (Pierce SnakeSkin^TM^ Pleated Dialysis Tubing, 10 kDa cutoff) in 50 mM HEPES-NaOH, pH 7.2, containing 1 mM DTT and 10% glycerol, concentrated using 2 ml volume capacity VivaScience concentrator of 10 kDa cut-off, and stored in 2-3 times usable aliquots at -80°C. Protein quantitations were all SDS-PAGE based, by making comparisons of coomassie blue-stained FtsZ bands with those from known amounts of bovine serum albumin.

Overexpression and purification of MsNDK and MsNDK-H117Q proteins

*E. coli* M15/ pQE30-MsNDK or pQE30-MsNDK-H117Q transformants [2] were grown to an OD_600 nm_ of 0.6 in LB broth (100 μg/ml of ampicillin and 25 µg/ml of kanamycin), followed by induction with 1 mM IPTG for 3 hrs. The cell pellet was washed with phosphate-buffered saline (pH 7.4) and suspended in the lysis buffer (100 mM Tris-HCl, pH 8.0, and 50 mM KCl) containing 1 mM PMSF. Sonication was carried out at 30 sec pulse for 15 cycles at 50% duty cycles to get clear lysate. Cell debris was removed by centrifugation at 12000 rpm for 20 min at 4ºC. The supernatant was loaded into a column containing 2 ml of Ni^2+^-NTA agarose slurry (Sigma), which was pre-equilibrated with the lysis buffer. The flow-through was recycled five times at 4ºC and the column was washed with the lysis buffer containing 20 mM imidazole to remove non-specific binding. Subsequently, the column was locked in elution buffer containing 250 mM imidazole for 30 min at 4ºC, and the protein was eluted using the same buffer. Near homogeneity preparations of overexpressed 6xHis-MsNDK or 6xHis-MsNDK-H117Q were obtained. The protein preparations were dialysed in lysis buffer containing 10% glycerol at 4ºC with three changes of 500 ml buffer. The protein preparations, subsequent to dialysis, were stored in aliquots at -75ºC for further use.

Production and affinity purification of polyclonal antibodies against MsNDK, MtNDK, MtFtsZ, and MsFtsZ

Polyclonal antibodies were raised against purified MsNDK, MtNDK, MtFtsZ, and MsFtsZ in rabbit (New Zealand white). Fourteen days after priming with the emulsion containing 200 μg of the antigen in Freund’s incomplete adjuvant, 100 μg of protein was used for 5 boosters of 7 days interval to get polyclonal antibody. Polyclonal sera raised against the protein were immuno-affinity purified, wherever mentioned, by using cyanogen bromide-activated sepharose beads, as described [6]. In brief, the polyclonal sera was added to the activated beads and kept in rotating shaker for 12 hrs at 4ºC. The unbound antibodies were washed and the protein-specific antibodies were eluted with 100 mM Glycine-HCl (pH 2.5) and the eluant was collected in a tube containing 1 M Tris-HCl, pH 8.0. The base-sensitive antibodies were eluted by passing freshly prepared 100 mM Triethylamine-HCl (pH 11.5). The eluant was collected in a tube containing 1 M Tris-HCl (pH 8.0) and stored in aliquots at -20ºC for subsequent use for immunoblotting as described [7].

Western blotting

Cytosol and membrane proteins were resolved using 12% SDS-PAGE. After fractionation, the proteins were electro-transferred to PVDF membrane, and the membrane was blocked for 3 hrs with blocking buffer (1x PBS buffer containing 0.1% Tween-20 and 5% skimmed milk). The PVDF membrane was incubated with anti-MsNDK or anti-MsFtsZ antibody (1:1000 dilution) for 3 hrs. The membrane was washed three times with wash buffer (1x PBS containing 0.1% Tween-20), incubated with anti-rabbit HRP secondary antibody (as per Sigma guidelines), and developed using ECL reagent (Sigma). For immunoblotting the pulldown assay samples, either mouse anti-GST antibodies (1: 1000) or anti-polyhistidine antibodies (1: 10000) was used. Antibodies (all of them rabbit polyclonal), were preincubated with the purified proteins at dilutions 1:100 for anti-MtNDK, anti-MtFtsZ and anti-GST before the addition of the cross-linker, DTSP.

**References**

1. Gupta P, Srinivasan R, Rajeswari H, Indi S, Ajitkumar P. In vitro polymerization of Mycobacterium leprae FtsZ OR *Mycobacterium tuberculosis* FtsZ is revived or abolished, respectively, by reciprocal mutation of a single residue. Biochemical and Biophysical Research Communications. 2008;368(2):445-52. doi: 10.1016/j.bbrc.2008.01.115. PubMed PMID: 18249190.

2. Arumugam M, Ajitkumar P. Histidine 117 in the His-Gly-Ser-Asp motif is Required for the Biochemical Activities of Nucleoside Diphosphate Kinase of *Mycobacterium smegmatis*. The Open Biochemistry Journal. 2012;6:71-7. doi: 10.2174/1874091X01206010071. PubMed PMID: 22888372; PubMed Central PMCID: PMC3414718.

3. Chopra P, Singh A, Koul A, Ramachandran S, Drlica K, Tyagi AK, et al. Cytotoxic activity of nucleoside diphosphate kinase secreted from *Mycobacterium tuberculosis*. European Journal of Biochemistry / FEBS Journal. 2003;270(4):625-34. PubMed PMID: 12581202.

4. Miroux B, Walker JE. Over-production of proteins in *Escherichia coli*: mutant hosts that allow synthesis of some membrane proteins and globular proteins at high levels. Journal of Molecular Biology. 1996;260(3):289-98. doi: 10.1006/jmbi.1996.0399. PubMed PMID: 8757792.

5. White EL, Ross LJ, Reynolds RC, Seitz LE, Moore GD, Borhani DW. Slow polymerization of *Mycobacterium tuberculosis* FtsZ. Journal of Bacteriology. 2000;182(14):4028-34. PubMed PMID: 10869082; PubMed Central PMCID: PMC94589.

6. Campbell DH, Luescher E, Lerman LS. Immunologic Adsorbents: I. Isolation of Antibody by Means of a Cellulose-Protein Antigen. Proc Natl Acad Sci U S A. 1951;37(9):575-8. PubMed PMID: 16589016; PubMed Central PMCID: PMC1063424.

7. Harlow E, Lane D. Antibodies: A Laboratory Manual. New York: Cold Spring Harbor Laboratory Press; 1988. pp. 313-315.
